# Representational similarity learning reveals a graded multidimensional semantic space in the human anterior temporal cortex

**DOI:** 10.1162/imag_a_00093

**Published:** 2024-02-22

**Authors:** Christopher R. Cox, Timothy T. Rogers, Akihiro Shimotake, Takayuki Kikuchi, Takeharu Kunieda, Susumu Miyamoto, Ryosuke Takahashi, Riki Matsumoto, Akio Ikeda, Matthew A. Lambon Ralph

**Affiliations:** Department of Psychology, Louisiana State University, Baton Rouge, LA, United States; Department of Psychology, University of Wisconsin, Madison, WI, United States; Department of Neurology, Kyoto University Graduate School of Medicine, Kyoto, Japan; Department of Neurosurgery, Kyoto University Graduate School of Medicine, Kyoto, Japan; Department of Neurosurgery, Ehime University Graduate School of Medicine, Ehime, Japan; Division of Neurology, Kobe University Graduate School of Medicine, Kobe, Japan; Department of Epilepsy, Movement Disorders and Physiology, Kyoto University Graduate School of Medicine, Kyoto, Japan; MRC Cognition and Brain Sciences Unit, Cambridge, United Kingdom

**Keywords:** Semantic memory, ECoG, temporal lobe, decoding, representational similarity analysis, RSA, representational similarity learning, RSL

## Abstract

Neurocognitive models of semantic memory have proposed that the ventral anterior temporal lobes (vATLs) encode a graded and multidimensional semantic space—yet neuroimaging studies seeking brain regions that encode semantic structure rarely identify these areas. In simulations, we show that this discrepancy may arise from a crucial mismatch between theory and analysis approach. Utilizing an analysis recently formulated to investigate graded multidimensional representations, *representational similarity learning* (RSL), we decoded semantic structure from ECoG data collected from the vATL cortical surface while participants named line drawings of common items. The results reveal a graded, multidimensional semantic space encoded in neural activity across the vATL, which evolves over time and simultaneously expresses both broad and finer-grained semantic structure among animate and inanimate concepts. The work resolves the apparent discrepancy within the semantic cognition literature and, more importantly, suggests a new approach to discovering representational structure in neural data more generally.

## 1 Introduction

If you encounter a wolf when walking home through a dark wood, your mind readily accomplishes some remarkable feats: it anticipates the animal’s likely behavior, perhaps slinking closer toward you; it assigns the thing a name, which you might shout to alert others (“wolf!”); and it directs you to change your own plans, maybe running back down the path. These feats arise from the human ability to discern conceptual structure—to realize that the wolf, despite its resemblance to friendly dogs in town, is nevertheless a quite different sort of animal.

This ability to represent and exploit conceptual structure is central to human semantic cognition. Such structure is *graded* in that similarities vary along a continuum: wolves are highly similar to coyotes, partially similar to elk, and quite distinct from birch trees or canoes. It is also *multidimensional* in that concepts vary along a myriad of independent components: wolves and dogs are similar in their shapes, parts, movements, and phylogeny, but different in their behaviors, habitats, and diets. To capture these properties of knowledge, computational approaches to semantics often represent concepts with *vector spaces*: the meaning of a word or image is expressed as a point in an *n*-dimensional space (or equivalently as an *n-*dimensional vector) such that the proximity between points expresses the similarity in meaning between the denoted concepts. The dimensions of the vector need not correspond to nameable conceptual components like *habitat* or *diet*; instead, they may define a space, with different concepts corresponding to different points in the space, and with the distances between points capturing the degree of semantic/conceptual relatedness between concepts (Frisby et al., 2023). Cognitive science and machine learning offer many techniques for estimating semantic vector spaces from natural language (Pereira et al., 2016), feature norms (McRae et al., 2005), or similarity-judgments (Hebart et al., 2020), and these methods have provided a critical empirical foundation for studying human conceptual knowledge.

The well-known “hub and spokes” theory of semantic representation suggests that the anterior temporal lobes (ATLs) encode a graded, multidimensional semantic vector space that expresses conceptual similarity structure for all concepts, extracted across all input and output modalities, and from our experiences of each concept across time (Jackson et al., 2021; Lambon Ralph et al., 2017; Patterson et al., 2007). This proposal has been useful for understanding patterns of semantic deficits arising from temporal lobe pathology in fronto-temporal dementia (Acosta-Cabronero et al., 2011; Hodges & Patterson, 2007; Lambon Ralph, 2014; Lambon Ralph et al., 2010; Rogers et al., 2004; Snowden et al., 1989), anterior temporal resection (Drane et al., 2008; Lambon Ralph et al., 2010, 2012; Rice et al., 2018; Schapiro et al., 2013), and herpes viral encephalitis (Gainotti, 2018; Lambon Ralph et al., 2007; Noppeney et al., 2007); stimulation and evoked response direct neurophysiological explorations (Abel et al., 2015; Chen, Shimotake, et al., 2016; Shimotake et al., 2015); the effects of transcranial magnetic stimulation in ATL and other parts of the cortical semantic system (Binney et al., 2010; Binney & Lambon Ralph, 2015; Jefferies, 2013; Lambon Ralph et al., 2009; Pobric et al., 2007, 2010); and a variety of behavioral phenomena in developing and mature cognition (Chen et al., 2017; Jackson et al., 2021; Rogers & McClelland, 2004, 2014).

Yet direct empirical tests of this proposal—representational similarity analysis (RSA) of functional imaging data collected while people perform semantic tasks on words or pictures—have not generally tended to support it. A recent review identifies 24 studies applying RSA to uncover semantic representations in the brain (Frisby et al., 2023); of these, 18 (75%) failed to identify semantic structure in the anterior temporal cortex (for the exceptions, see Bruffaerts et al., 2013; Devereux et al., 2018; Fairhall & Caramazza, 2013; Martin et al., 2018; Peelen & Caramazza, 2012). Many of these studies instead find that semantic structure is encoded in brain areas not otherwise thought to be critical to semantic representation, including posterior cortical regions (Connolly et al., 2012), inferior and superior frontal and motor cortex (Carota et al., 2017; Wang et al., 2017), the left pars triangularis (Liuzzi et al., 2017), the right superior parietal cortex (Wang et al., 2017), the insula and occipeto-parietal cortex (Kivisaari et al., 2019), and the posterior cingulate cortex (Fairhall & Caramazza, 2013). Thus, RSA studies often yield results that seem puzzling given the broader literature, finding that semantic structure is encoded in many areas throughout cortex but not in the ATL.

The frequent failure of RSA to find semantic structure in ATL may reflect limitations of fMRI, which, without specialized acquisition protocols, can yield poor signal in ventral aspects of this brain area (Halai et al., 2014, 2015). An important study by Chen, Shimotake, et al. (2016) suggests, however, that this is not the full story. The authors collected intracranial grid electrode voltages (ECoG) from the surface of ventral anterior temporal lobes (vATL) while participants named line drawings of familiar items, then conducted a searchlight-based semantic RSA from these data. Consistent with the semantic-hub model, they found an anterior fusiform area where similarities in the evoked neural response correlated significantly with the target semantic similarities. Critically, however, they further showed that the evoked neural similarities correlated *equally well* with a binary target matrix that only encoded whether a stimulus was animate or inanimate. This result is consistent with an alternative view that, while ECoG in vATL may express a coarse binary animacy distinction, it does *not* otherwise encode graded or multidimensional semantic structure within or between these domains. ECoG is not affected by the magnetic field inhomogeneities that affect fMRI in vATL, so the finding is not easily attributable to poor signal or other data artifacts.

Motivated by these observations, this paper considers an alternative hypothesis about the discrepant findings for RSA versus the broader literature: that it arises because RSA, as typically practiced, is not well suited to finding *graded multidimensional* vector spaces of the kind the ATL is hypothesized to encode. This may seem surprising, since RSA was developed specifically as a tool for finding cognitive similarity structure in neuroimaging data (Kriegeskorte, Mur, & Bandettini, 2008)—yet, as we will demonstrate, the reliance of the approach on correlation significantly limits the kinds of signal it can detect.

Study 1 considers how RSA constrains what can be discovered about neuro-semantic representations, by applying the approach to simulated data where the target signals are designed to encode elements of structure in a true semantic similarity matrix derived from feature-listing data. The results illustrate how and why RSA can both miss real signal and produce positive results with the potential to mislead. Study 2 then introduces a different approach to neural decoding, *representational similarity learning* (RSL; Oswal et al., 2016), that can remediate these issues by making explicit which aspects of the target structure can be successfully decoded, and by operationalizing hypotheses about the neurosemantic code directly within the decoding model. Study 3 extends the RSL approach to the analysis of very large neural datasets and applies it to investigate graded multidimensional semantic structure in ECoG data recorded from the surface of human vATL—the same data for which RSA data failed to detect graded, multidimensional, and cross-domain semantic structure in a prior study (Chen, Shimotake, et al., 2016). We empirically compare results yielded by RSL versus RSA on these data, with results that resolve the contradiction in the literature and suggest a new path for multivariate neural decoding more generally.

## 2 Simulation Study 1: Evaluating RSA

Simulation 1 evaluated whether the results yielded by RSA reliably indicate whether a set of neural measurements encode graded, multidimensional semantic similarity structure of the kind typically sought in functional imaging studies. By *graded*, we mean that the measurements encode varying and continuous degrees of similarity between items, rather than discrete or categorical distinctions between items. By *multidimensional*, we mean that the measurements encode variation along more than one orthogonal component of a representational space. Though the general approach is well known, its limitations may be less familiar, so we begin with a brief overview of the method and some of the challenges it faces when seeking graded, multivariate structure.

RSA aims to find sets of neural features—voxels, electrodes, or other measurements of neuro-physiological activity—whose responses to various cognitive events (e.g., the perception of a stimulus) jointly encode an independently-measured target structure. Typically, the target structure is a *representational similarity matrix* (RSM; sometimes called a representational dissimilarity matrix or RDM) in which the rows and columns correspond to the different stimuli in an experiment and the entries indicate the cognitive/representational similarities between stimuli. For semantic representation, entries in the target RSM indicate similarity of meaning among pairs of concepts as estimated from behavioral or corpus data. To determine if responses in a set of neural features (e.g., voxels, electrodes, EEG sources, etc.) encode the target similarities, the experimenter estimates the pattern of neural activity evoked over features by each stimulus and computes the pairwise similarities between these to create a *neural similarity matrix* (NSM). Correlations between the RSM and NSM are computed separately for each participant, and brain regions where these are reliably greater than zero are interpreted as encoding the target structure.

This approach faces at least five challenges when used to find *graded*, *multidimensional* structure in neural codes:

1. *Discrete versus graded structure*: reliable correlations with a continuous-valued target RSM can arise even if the neural response is discrete or categorical. Thus, a positive result does not indicate that the neural response encodes *graded* structure even if such structure is present in the target RSM.
2. *Unidimensionality*: the correlation between RSM and NSM is inherently unidimensional. Thus, a positive result on its own does not provide evidence of multidimensional structure in the neural response.
3. *A priori feature selection*: the experimenter must decide ahead of time which neural measurements to use when computing the NSM (e.g., an ROI or a searchlight). If the informative features fall across different ROIs or searchlights or are intermixed with many non-informative features within an ROI/searchlight, the approach can fail to discover them.
4. *Equal importance*: all selected neural features are equally weighted when computing the NSM. If the informative neural features are sparse or vary in signal strength, equal weighting of all features may lead to a null result. Thus, a null result does not indicate that the selected neural features carry no target information.
5. *Masked dimensionality*: reliable correlations between RSM and NSM can be driven by a subset of the RSM structure or items, or even by one or two extreme values. Thus, a positive result does not imply that all components of the target RSM are encoded.

Simulation 1 assessed how these characteristics of RSA will influence discovery of neuro-semantic representations by applying the approach to synthetic data designed to encode different aspects of a real semantic similarity matrix.

### 2.1 Methods

The target RSM was computed from semantic feature norms for 100 items, half animate and half inanimate, collected in prior work (Dilkina & Lambon Ralph, 2013). The feature vectors for each item were aggregated in a matrix with rows indicating words, columns indicating features, and binary entries indicating whether the referent of the word possesses that feature. Matrix columns were mean-centered, and the RSM was computed as the cosine similarity for all pairs of row vectors.

To understand the latent structure of the RSM, we used singular value decomposition (SVD) to extract three components accounting for 90% of the variance in the full matrix (81.1%, 4.4%, 4.0%, respectively). SVD is a common matrix decomposition algorithm that reduces a matrix to a small number of orthogonal components ordered by the proportion of variance they explain. After applying it to the RSM, each of the 100 items in the set receives a coordinate position along the three orthogonal components.

**Fig. 1.**
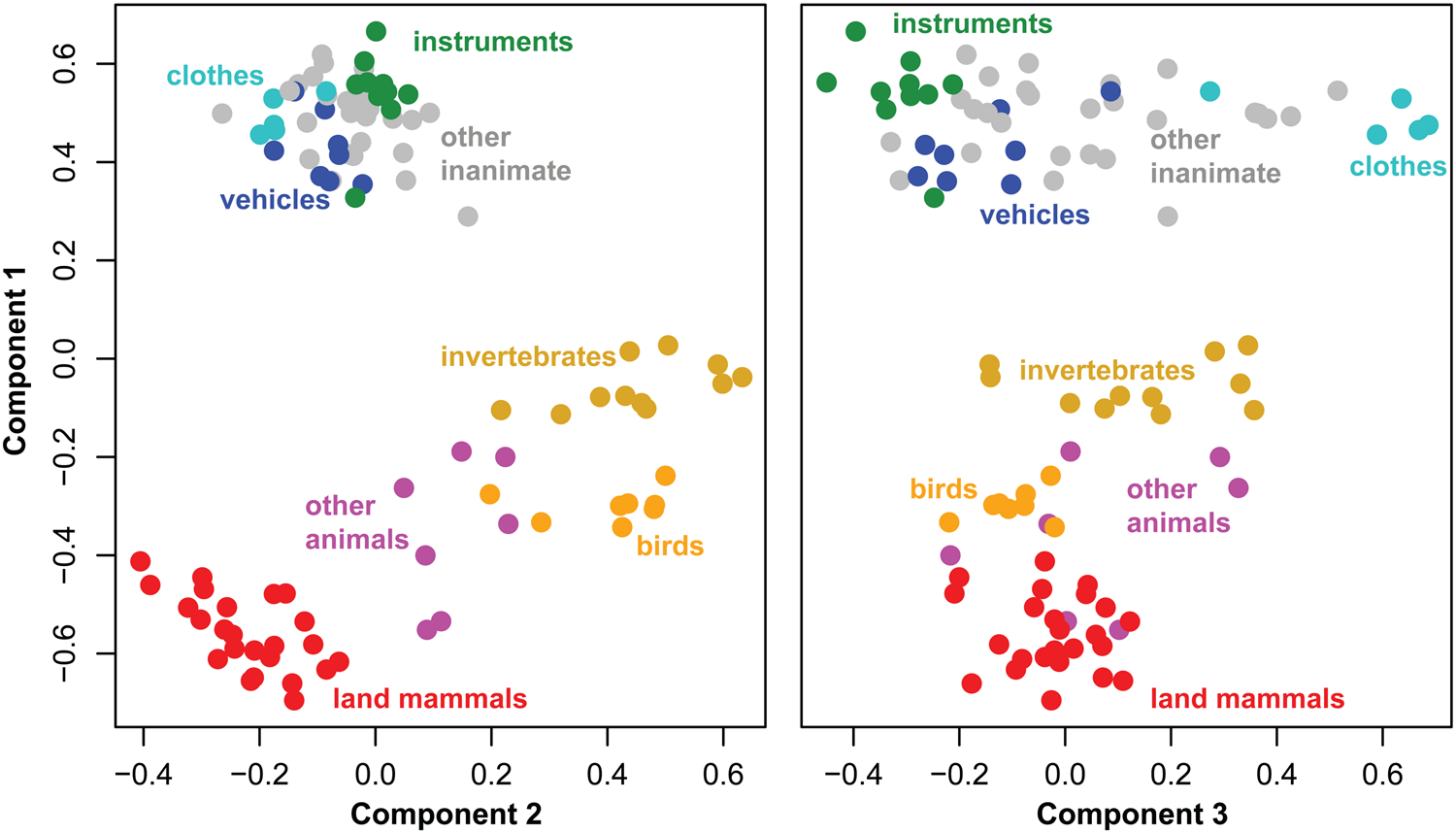
Coordinates of the 100 stimuli in the three-dimensional singular-value-decomposition of the semantic similarity matrix. Colors show category membership within each domain. Component 1 not only separates animate from inanimate items, but also separates animate subcategories. Component 2 largely separates animate subcategories, while component 3 largely separates inanimate subcategories.

Figure 1 shows the coordinates for all 100 items on each latent dimension, color coded by semantic category (see Supplement A for full list of items and how they were categorized). The decomposition reveals graded semantic structure along each dimension. The first not only separates animate from inanimate items but also individuates subcategories in each domain. The second not only strongly differentiates subcategories of animals but also weakly distinguishes inanimate subcategories. The third differentiates the inanimate items, though the resulting spread is less “clumpy” than within animate items as commonly found with such data (Lambon Ralph et al., 2007). From the SVD, it is clear that the semantic vector space is both multidimensional and graded by the definitions offered earlier.

The simulation assessed what results RSA would yield when applied to a set of signals designed to encode elements of this underlying semantic structure, when those signals are perturbed by noise and embedded in a population together with other signals that do not code semantic information (as in brain imaging data). Specifically, we created five simulated datasets, each capturing a different aspect of semantic structure in the target RSM. Each dataset contained simulated responses of 24 features to the 100 items, as a simple model analog of the responses of, for instance, voxels within an ROI. To capture the fact that a given ROI may contain both signal-carrying and uninformative voxels, half of the features encoded true semantic information about the target items while half adopted random values sampled from a uniform distribution in the same range.

**Fig. 2.**
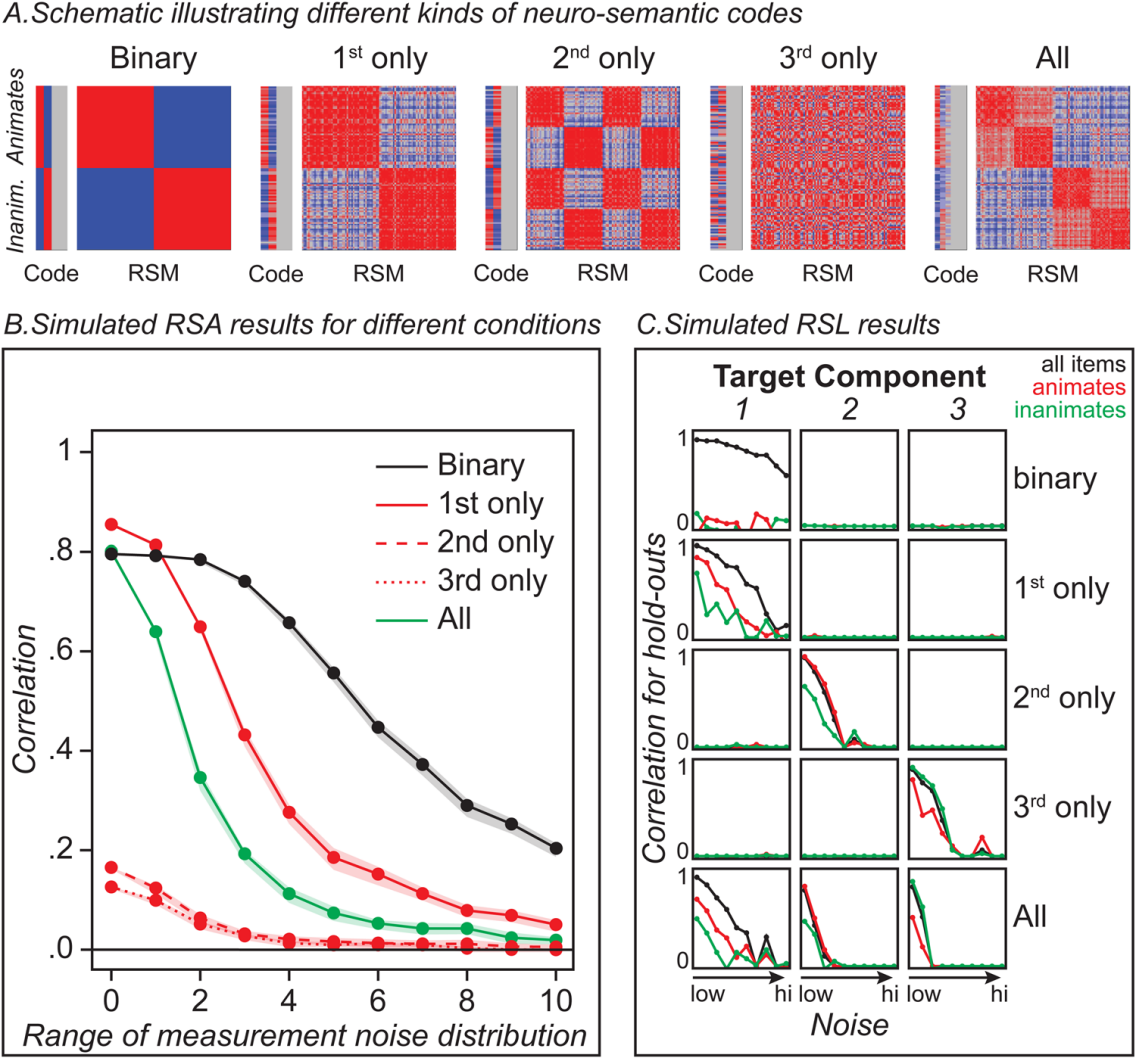
Simulation results. (A) Each plot illustrates different ways that a neural response might encode some aspects of a target RSM, schematized in the rightmost plot (“All”). Vertical bars show the hypothesized responses of neural features to various animate and inanimate stimuli (red more active; blue less active; gray random) while the squares show the neural similarity matrices that would then result (red high similarity, blue low). All conditions encode some components of the full RSM and so should be detected by a multivariate method seeking semantic structure. (B) Curves showing the expected results of standard RSA when used to decode real semantic structure from the target RSM under different hypotheses about the neural code and increasing amounts of measurement noise. Dots show the mean correlation, while ribbons show the 95% confidence interval. The approach can yield robust results when the neural signal is discrete and/or unidimensional, even under substantial noise, and can yield weak or null results when the neural signal faithfully encodes weaker components of the RSM, even under low-noise conditions. (C) Results of RSL applied to the same simulated data, showing the correlation between true and predicted coordinates for held-out items on each latent component of the target matrix (columns) at the same 11 levels of noise as in the RSA, for the five different simulation conditions (rows), and computed across all items (black lines), animate items (red), or inanimate items (green). RSL shows a positive result in each case, identifies which components of the matrix are present in the neural code, and reveals reliable within-domain decoding only when the neural code expresses continuous similarity structure.

The five datasets varied in which aspects of the target matrix they encoded, as illustrated in the schematic plots in Figure 2A. In the *binary condition*, all signal-carrying features adopted one state (-1 or 1) for animate items and the opposite state for inanimate items—thus, the code was both unidimensional and discrete (i.e., non-graded). In three *one-D conditions*, all signal-carrying features only encoded a single latent component of the target RSM—either the first, second, or third singular vector as shown in Figure 1. Thus, each encoded *graded* similarity structure (the distances between items were continuous, not binary/discrete) but only *one* dimension of variation. In the *full structure condition,* the signal-carrying features jointly encoded all three latent components of variation in the target RDM, with four features dedicated to each of the three components. Thus, the code was both graded and multidimensional.

For each signal condition, we distorted the response of each feature to each stimulus with measurement noise sampled independently from a uniform distribution centered on 0, generated a simulated NSM, then computed the correlation between the simulated NSM and target semantic RSM. The varying levels of noise allowed us to investigate RSA behavior across a wide range of signal-to-noise ratios. This procedure was conducted 20 times at each of 11 increasing noise levels. We then computed, across runs at each noise level, the mean and 95% confidence intervals of the Pearson correlation between simulated NSM and RSM.

### 2.2 Results

The results are shown in Figure 2B. The binary code showed robust correlations across the full noise-spectrum, illustrating that RSA with a graded and multidimensional target RSM can yield a positive result even when the neural code is neither continuous nor multidimensional. Amongst the one-D codes, RSA showed a strong positive result if the simulated neural response encoded the first singular vector of the target matrix, but much weaker results that decayed to zero with increasing noise when the neural response encoded other components. The weak results for components 2 and 3 arise simply because the raw similarities encoded in the target RSM are most strongly determined by the first component. Simulated NSMs that encode just the second or third component thus express similarities that do not correlate strongly with the pairwise similarities encoded in the target matrix. Thus, RSA can yield weak/null results even when the features under consideration *do* reliably encode semantic information that is orthogonal to the primary component of the RSM. Throughout the noise range assessed, RSA yielded the most reliable results when the simulated features encoded a binary domain distinction or just the first component of variation. For these scenarios, all signal-carrying features encode the same information, so noise “cancels out” across features and the signal remains robust. When the same number of features encode three orthogonal components, fewer resources are dedicated to each, and corruption from noise more seriously degrades the signal—so that, counter-intuitively, the approach yielded less reliable results when the simulated features encode all three dimensions of the target matrix.

### 2.3 Discussion

The simulation shows that, when decoding semantic similarity structure of the kind captured by semantic feature norms, RSA can yield a positive result when the neural response is discrete and/or unidimensional, *even* though the target matrix is continuous and multidimensional. Additionally, it can yield weak or null results when the neural response *does* encode underlying dimensions of the target matrix beyond the strongest. Thus, the simulation serves as a demonstration that RSA, as typically deployed, may not reliably indicate whether a set of neural signals encode the graded, multidimensional semantic structure existing in a target RSM. Of course, the specific pattern of results obtained will depend upon the ratio of informative to uninformative features within the selected set and the amount of noise perturbing the signal, in addition to the structure encoded by the informative features.

One response to these challenges may be to consider the degree of correlation between a given NSM and multiple different RSMs, each encoding a different kind of structure. Yet, simulation 1 suggests that this approach remains limited. Recall that Chen, Shimotake, et al. (2016) found that an ECoG-based NSM correlated equally well with an animacy-based binary similarity matrix as with a full continuous and multidimensional matrix. The rationale for the comparison was the expectation that, if the NSM encodes the full similarity structure, it should show a higher correlation with the target semantic matrix than with the binary matrix. Figure 2B shows that this is not necessarily so—in fact, the binary code showed as good or better correlation with the target semantic matrix than did the full (continuous, multidimensional) code. Thus, the comparison of fits with the two different target matrices did not help to resolve the nature of the underlying neural code.

Or, consider the interesting findings of Clarke and Tyler (2014), who used RSA to seek areas encoding semantic structure both across and within superordinate domains (such as “animals” and “vehicles”). When the target matrix included all items from all domains, the analysis identified the vATL as encoding semantic structure. When limited only to items within one semantic domain, however, the vATL did not appear to encode such structure. The finding could mean that vATL only encodes discrete distinctions between superordinate semantic domains, analogous to the binary condition in the simulation—but the same result could also be obtained even if the vATL does encode within-domain structure, since such structure is mainly encoded, within the semantic target matrix, by components orthogonal to the primary dimension.

More sophisticated use of RSA may overcome some of these difficulties in interpretation; for instance, the researcher might carefully select stimulus items to ensure that multiple orthogonal components of a representational space are equally strongly expressed in the RSM, or to ensure that different target representational spaces are maximally differentiated by the selected stimulus items. Indeed, one of the few RSA studies to report semantic representational structure in ATL adopted this approach, ensuring that semantic and visual similarity structure were completely deconfounded in their stimuli and then comparing results for these two differently-structure target matrices (Martin et al., 2018). While such studies are elegant and informative, they are also challenging to design and may prevent researchers from exploring fuller or more naturalistic distributions of stimuli. For these reasons, we were motivated to consider an alternative approach to discovery of graded multidimensional representations in neural data.

## 3 Simulation Study 2: Decoding with RSL

The approach we developed is called *representational similarity learning* (RSL; Oswal et al., 2016). Rather than using pairwise similarities in the RSM as the target values for correlation, RSL first decomposes the matrix into orthogonal latent components, effectively re-representing each stimulus item as a point in a low-dimensional semantic space as shown in Figure 1. It then uses linear regression to fit *decoding models* that predict the coordinates of each item along each dimension from their evoked neural responses. Simulation 2 assessed whether this approach can reveal the graded and/or multidimensional structure obscured by RSA.

### 3.1 Methods

Using the same simulated neural signals from Simulation 1 as predictors, we fit ordinary least-squares (OLS) regression models to predict coordinates of each stimulus along each of the three latent semantic dimensions shown in Figure 1. For each simulated feature set and each of 11 increasing levels of uniformly distributed noise (i.e., the same noise manipulation as for Simulation 1), each example was assigned to 1 of 10 mutually exclusive test sets such that each test set had 5 animate and 5 inanimate items. Holding out each test set in turn, regression models were fit to the remaining 90% of the examples (the training set). The model that was fit to the training set predicts coordinates for the 10% of examples withheld as the test set. After iterating over the 10 test sets, a holdout prediction exists for every item. These were concatenated and correlated with the target embedding one dimension at a time. This process was repeated 20 times with different samples of noise. Holdout accuracy for each condition and noise level was estimated as the mean over simulation repetitions.

Multidimensional structure in the neural code should be revealed by reliable prediction of coordinates along more the one latent dimension. To assess whether the neural code captures graded similarity, we evaluated how well the fitted model predicted coordinates among just the animate items or just the inanimate items. If the neural signal only categorically differentiates animate from inanimate stimuli, then predicted and true values should correlate significantly when models are evaluated on all items but should *not* correlate among just the animate or just the inanimate items considered separately. Reliable intra- and inter-domain correlations between true and predicted values thus indicate that the neural code captures a graded degree of similarity structure.

### 3.2 Results

Figure 2C shows the mean correlation between true and predicted coordinates for held-out items on each of the three target components (columns), for the five different neural coding scenarios (rows), and computed across all items (black lines), animate items (red), or inanimate items (green). The pattern of results reveals the information encoded in each simulated neural response. With the binary neural signal (top row), predicted and true coordinates correlated strongly for models fit to all items but not separately for animates versus inanimates. With continuous one-D signals, strong correlations were observed for models fit to all items and within each domain separately, but only for the dimension encoded by the neural response. When the neural signal encoded graded multidimensional structure, reliable correlations were observed on all three latent components, for models fit to all items, and for models fit separately to each domain. Thus, in simulation, the pattern of results uniquely revealed whether the neural signal encodes graded or discrete structure, and which semantic dimensions it captures.

### 3.3 Discussion

In contrast to the RSA simulation, RSL yielded a different, diagnostic pattern of results for each condition, indicating which aspects of the semantic target matrix were encoded within the simulated neural responses. The discreteness of the binary code was revealed by reliable prediction across domains and no reliable prediction within domain. In the 1D neural-structure conditions, RSL showed reliable prediction only on the latent dimensions encoded by the simulated neural signal, and not on the other dimensions. Reliable decoding both within and between domains in such conditions indicated, in contrast to the binary condition, that the signal is graded rather than discrete. When the simulated neural signal encoded all three semantic dimensions, RSL showed reliable decoding of each. Thus, by considering decoding accuracy on orthogonal components of the target matrix, RSL can test whether the neural code expresses multidimensional structure; and by considering decoding accuracy separately for different semantic domains, it can test whether the code is graded.

## 4 Study 3: Decoding Semantic Structure from ECoG Using RSL

Study 3 evaluated whether neural signals in vATL encode a graded, multidimensional and domain-general semantic space by using RSL to decode semantic structure in ECoG data collected in a prior study of object naming (which employed the same 100 items used in the simulations; Chen, Shimotake, et al., 2016). This dataset is especially useful in the current context for two reasons.

First, ECoG avoids some of the limitations of non-invasive imaging methods that can make structure in vATL difficult to decode. One recent study suggested that the neuro-semantic code for animacy in vATL changes rapidly and nonlinearly over the course of stimulus processing so that techniques sacrificing either spatial or temporal resolution, including EEG, MEG, and fMRI, may obscure important signals (Rogers et al., 2021). Additionally, magnetic field inhomogeneities make it difficult to resolve clear BOLD signal in vATL without special acquisition sequences (Halai et al., 2014, 2015). ECoG provides much better spatial and temporal resolution of neurophysiological signals at the surface of the cortex and does not suffer from the signal-detection issues that challenge fMRI, so any failure to discover fine-grained / multivariate semantic structure in the data cannot reflect these limitations.

Second, the results of the original RSA study (Chen, Shimotake, et al., 2016) suggest that the measured signals, though reliably differentiating animate from inanimate items, do *not* encode finer grained or multidimensional semantic structure. Specifically, the authors compared the correlation of the stimulus evoked NSM to two different target matrices: one encoding the full graded and multidimensional semantic similarities among stimuli, and a second encoding just the binary distinction between animate and inanimate items. If the vATLs encode graded multidimensional semantic structure, one might expect the NSM to correlate more strongly with the full target matrix than with the binary animacy matrix. Instead, the authors found statistically significant and *equally strong* correlations with both target matrices. One interpretation of this finding is that the measured neural signals do *not* in fact encode graded or multidimensional semantic structure, but only serve to discretely differentiate animate and inanimate items—a conclusion also consistent with Clarke & Tyler’s (2014) RSA results described earlier. The data thus allow us to test an alternative hypothesis: that the measured signals *do* encode a graded, multidimensional, and domain-general semantic space, in a manner that is invisible to RSA. If so, RSL should reliably decode variation along multiple orthogonal components of the target semantic matrix, considering all items together as well as within animate and inanimate domains considered separately.

Applying RSL to ECoG or any other brain imaging data, however, faces an immediate challenge: such technologies produce many more spatiotemporal neural measurements than there are stimuli, so regression models predicting stimulus characteristics from neural data are ill-defined (i.e., there exist infinite solutions that can perfectly predict outcomes on training data even from random input data). To find a unique fit without a priori feature selection, the regression must be *regularized* to satisfy some additional constraint that will guarantee a single unique solution for model fitting. For instance, the weight optimization might jointly minimize prediction error and the size of the model coefficients, measured as the sum of their absolute values (the L1 norm). Since this sum can be minimized by placing zero coefficients on many predictors, this form of regularization promotes a sparse solution in which only a few predictors have non-zero coefficients in the final model (e.g., Rogers et al., 2021). Other regularizers (such as the L2 norm or the elastic net) enforce different constraints on model fitting, and thus lead to other solutions when applied to the same dataset (Cox & Rogers, 2021).

In this sense, the selection of a regularization function amounts to a prior hypothesis stipulating how the neural signal is likely to be structured. As recently argued in Frisby et al. (2023), the choice of regularizer should be informed by explicit hypotheses about the nature of the signal to be decoded—in this case, a hypothesis about how neurophysiological signals measured by surface electrodes in the brain might encode a graded, multivariate semantic space.

To that end, we adopted a regularization function called the *group-ordered-weighted LASSO* (grOWL) loss (Oswal et al., 2016) that was designed in prior work to capture three theoretically motivated assumptions about the structure of neural representations. First, it assumes the signal is *sparse*: of all measurements taken, only a relatively small proportion encodes target information of interest, so the regularizer should place zero coefficients on many predictors (as with L1 regularization). Second, it assumes *redundancy* in the true signal: neural populations that do encode target information will be correlated in their activity patterns over items, so the regularizer should spread similar coefficients across correlated signal-carrying populations (similar to L2 regularization). Third, it assumes that signal-carrying populations are unlikely to be “axis-aligned” with the target semantic space (i.e., responding only to variation along a single semantic dimension) but more likely encode multiple, varying “directions” within the space. That is, each neural population likely carries information about an item’s coordinates along multiple dimensions of the target space. We will refer to this as the *spanning* assumption, since the selected neural features span the target representational space but are unlikely to only encode distinct, individual dimensions. To enforce this assumption, the regularizer should prefer solutions where coefficients on a given neural feature are either all zero (the feature does not carry any information) or all non-zero (it predicts some variation along all target dimensions).

Oswal et al. (2016) showed that all three assumptions can be captured in a single convex loss function, and also provided analytic guarantees and an example application on an fMRI dataset. We describe the logic briefly here; Supplement B provides a formal description of the decoding model and grOWL regularizer, including a definition of the loss and explanation of how it encourages these properties in the decoding matrix.

**Fig. 3.**
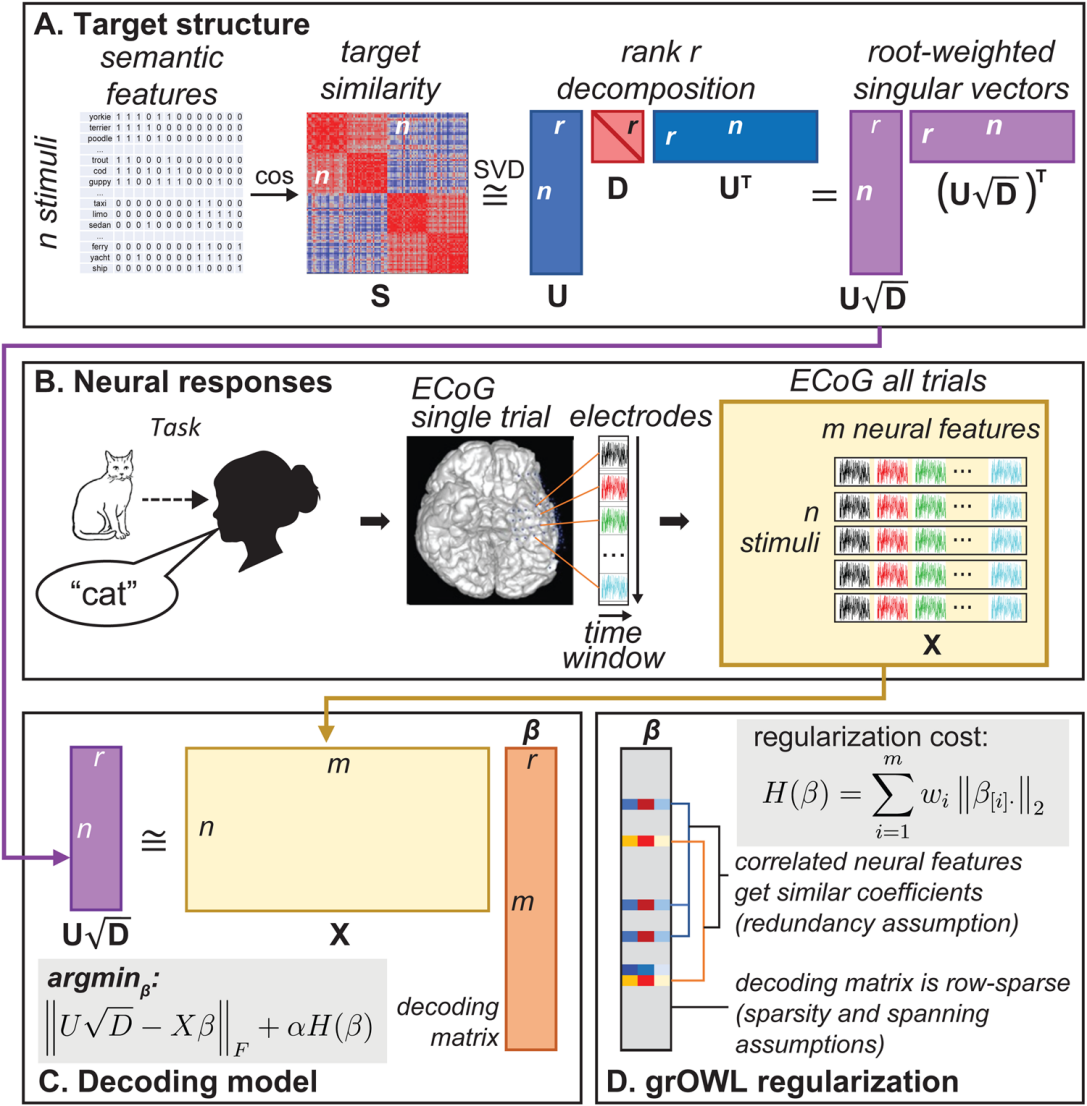
Representational similarity learning. (A) To generate the target structure for decoding, semantic feature vectors for each of *n* items are mean-centered and converted to a cosine similarity matrix ${\text{S}}_{n\times n}$ of approximate rank r. This is decomposed into matrix ${\text{U}}_{n\times r}$ containing r orthogonal singular vectors and diagonal matrix ${\text{D}}_{r\times r}$ containing singular values for each vector. The product $\text{U}\sqrt{\text{D}}$ is an *nxr* matrix whose columns contain *r* root-weighted singular vectors that are the target of the decoding model. Note that the product of this matrix with its transpose provides an estimate of the original matrix ${\text{S}}_{n\times n}$. (B) Neural responses in a picture-naming task are recorded for each stimulus as intra-cranial voltage potentials collected at 1000 Hz over electrodes implanted in vATL. Data are concatenated across electrodes to create a single *m*-dimensional “neural feature vector” for each stimulus, where *m* is the total number of electrodes times the size of the decoding window in milliseconds. All such vectors are compiled into a neural feature matrix ${\text{X}}_{n\times m}$ which contains the predictors for the decoding model. (C) The target structure $\text{U}\sqrt{\text{D}}$ is then modeled as the product of the neural feature matrix X and a decoding matrix of regression coefficients β whose values are chosen via gradient descent to minimize prediction error plus the regularization penalty H(β) scaled by the weighting parameter α. (D) Definition of the group-ordered-weighted LASSO (grOWL) regularization function and illustration of the structure it promotes in the decoding matrix.

Rather than fitting a separate regression model for each latent component of the target RSM (as in the simulation), we instead predict all latent components (Fig. 3A) simultaneously by estimating the parameters of a *decoding matrix*β (Fig. 3C) with regularized multitask regression. Suppose the coordinates of *n* stimuli on *r* latent semantic dimensions are stored in matrix ${\text{U}}_{n\times r}$ while the responses of *m* neural features to the stimuli are stored in matrix ${\text{X}}_{n\times m}$. RSL models the entries in U as the matrix product of the neural activations in X and the decoding matrix $\beta_{m\times r}$: U=Xβ. Each row of β corresponds to one neural feature (voxel, electrode, etc.), and each column encodes weights for each neural feature when predicting stimulus coordinates on the corresponding dimension of the target matrix U. Thus, the assumptions about neural signal just listed can be formalized as constraints on the structure of the decoding matrix β (Fig. 3D). To capture the sparsity and spanning assumptions, β is constrained to be *row-sparse*: most rows have all zero values (sparsity), and the rest have all non-zero values (spanning). To capture the redundancy assumption, highly correlated and signal-carrying neural features receive similar row-vectors in β (Fig. 3D). The precise degree of sparsity and redundancy in the decoding matrix is controlled by hyper-parameters that are tuned via cross-validation. Figure 3 shows the full workflow decoding semantic structure from ECoG data.

With this overview, we can consider how RSL can be applied to assess whether neural data encode a multidimensional and graded similarity space. Parameters in β are estimated for a batch of training data using regression regularized with the grOWL loss. The decoding model is then applied to predict the coordinates of held-out items along each dimension in the target representation space. If predicted and true coordinates for held-out items correlate reliably on more than one dimension, the neural signal must encode multidimensional structure. To assess whether the neural code captures graded similarity, we additionally evaluate the regression models separately for animate and inanimate items, as in the simulation. If the neural signal only categorically differentiates animate from inanimate stimuli as suggested by the prior RSA analysis in Chen, Shimotake, et al. (2016), then predicted and true values should correlate significantly across all items, but not for animate or inanimate subsets considered separately. Observation of both inter- and intra-domain correlations thus indicates that the neural code captures a graded degree of similarity structure. Finally, to assess how well the decoder recovers the target semantic similarities, the predicted coordinates for held-out items on all three latent dimensions can be used to estimate semantic distances between stimulus pairs. These estimates can then be correlated with true semantic distances, similar to standard RSA.

### 4.1 Methods

We applied this approach to the same ECoG dataset where prior work using RSA found no evidence for graded multidimensional structure (Chen, Shimotake, et al., 2016). The study was approved by the ethics committee of the Kyoto University Graduate School of Medicine (No. C533), and participants provided written informed consent. The dataset contains voltages measured from platinum subdural grid electrodes (inter-electrode distance 1 cm and recording diameter 2.3 mm; Ad-Tech, WI) implanted in the surface of left (8) or right (2) vATL in 10 patients undergoing preparation for intractable epilepsy (9) or brain tumor (1) while they named line drawings depicting the 100 items described in Study 1. Electrodes were implanted in the right hemisphere for two patients where WADA testing did not clearly indicate left-lateralization of language.

Each patient had between 6 and 32 electrodes (mean of 20) covering vATL. All patients with epilepsy had seizure onset zones outside the anterior fusiform region, except one patient for whom it was not possible to localize the core seizure onset region. Data were sampled at 1000 Hz (eight patients) or 2000 Hz (two patients) with a band-pass filter of 0.016–300 (eight patients) or 0.016–600 Hz (two patients). The dataset included, for each stimulus, voltages measured at each electrode over a 1 s window from stimulus onset and downsampled to 100 Hz. Thus, for a participant with 20 electrodes, each stimulus was associated with a 2000-element vector of voltages (20 electrodes x 100 time points). The ECoG data were preprocessed exactly as in Chen, Shimotake, et al. (2016).

#### 4.1.1 Stimuli and procedure

One hundred line-drawings (50 animate and 50 inanimate items) were obtained from previous norming studies (Morrison et al., 1997; Snodgrass & Vanderwart, 1980). See Chen, Shimotake, et al. (2016) for a complete list. Animate and inanimate stimuli were matched on age of acquisition, visual complexity, familiarity, and word frequency, and had high name agreement. Each stimulus was presented on a computer screen for 5 s, one after another with no interstimulus interval, once during each of four sessions (four times in total). Each session proceeded in a different random order. Participants were instructed to name each item as quickly and accurately as possible. Participants were video and audio recorded during the experiment. Video was used to monitor eye fixations and general attention to the task. The mean response latency was 1190 ms.

#### 4.1.2 Semantic similarity structure and dimensionality reduction

The same target RSM and three-dimensional embedding used in the simulated analysis are reused for the ECoG analysis.

*ECoG data preprocessing.* Preprocessing was performed in MATLAB. Data were downsampled to 100 Hz by averaging measurements within 10 ms boxcars. A prior study applying pattern classification to these data found near identical results when analyzing raw voltages versus voltages referenced to the electrode beneath the galea aponeurotica or to the scalp electrode on the mastoid process contralateral to the side of electrode implantation (Rogers et al., 2021). For this reason, and because the location of the reference electrode varied across patients, we analyzed all voltages without referencing. For each stimulus, we retained data for 1000 ms from stimulus onset. While the trial epoch may include the onset of articulation toward the end, the critical results cannot reflect such motor activity since all key phenomena are observed early in the epoch. Baseline correction was not performed. The mean voltage at each electrode for each stimulus was computed across the four repetitions. Voltages from different electrodes were concatenated into a row vector and arranged in a neural data matrix with each row containing the vector of voltages for one stimulus sampled from multiple electrodes over time. We rejected columns and then rows where the marginal mean was more than five standard deviations from the grand mean to censor extreme outliers.

#### 4.1.3 Model fitting and evaluation

RSL decoding models regularized with either grOWL or the L1-norm were fit and analyzed in MATLAB v9.5 (Mathworks Inc, 2018) using the Whole-brain Imaging with Sparse Correlations (WISC) toolbox (Cox, 2022), which implements the approach as a multitask variant of group ordered-weighted L1-regularized regression (grOWL; Oswal et al., 2016; Oswal & Nowak, 2018).

Fitting a decoding model regularized with grOWL requires two hyper-parameters that govern the pressured strength with which the decoding matrix is to be row-sparse (λ) with similar weights across correlated neural features (ω). We chose values for λ and ω via 10-fold nested cross-validation for each model fit. The 100 stimuli were randomly divided into 10 subsets, each containing 5 animate and 5 inanimate items. On each of the 10 folds, one of the 10 subsets was held out (outer-loop holdout). The remaining nine sets were used to search for good hyperparameter values, involving a second “inner” cross-validation loop.

On each inner-loop fold, one of the nine remaining sets was held out and a decoding model was fit to the remaining eight sets using a specified pair of hyper-parameter values. The fitted model’s prediction error, defined as the Frobenius norm of true versus predicted semantic coordinates, was evaluated on the inner-loop holdout set. This procedure was repeated once for each of the nine inner-loop holdouts before averaging the prediction error across folds to estimate the decoder’s performance with that hyperparameter configuration. Many different hyperparameter configurations were evaluated in the inner loop using the Hyperband procedure (Li et al., 2018). The best-performing configuration was then used to fit a model to all examples not in the outer-loop holdout set. This tuned model was used to predict semantic coordinates for the outer-loop holdout set, thus completing one iteration of outer-loop cross-validation. The whole procedure was then repeated for the remaining nine outer-loop holdout sets. Across folds, the procedure generated out-of-sample predicted coordinates for all 100 items. These final predicted coordinates, computed separately for each time-window in each participant, were the primary data evaluated in the results.

#### 4.1.4 Statistical thresholding with permutation testing

Our analyses closely followed the simulations: correlations between predicted and true coordinates on each dimension were computed for all 100 stimuli, just the 50 animate items, or just the 50 inanimate items. Assessing model accuracy via correlation standardizes means and variances of the target and predicted vectors to focus on just their covariance. This is especially critical when evaluating animate and inanimate items separately. However, cross-validated correlation has a negative bias when evaluating held-out items (see Zhou et al., 2017 and Supplement C); a null hypothesis of zero with a t-distributed sampling distribution cannot be assumed. Thus, we determined statistical reliability at the group level by constructing an empirical null distribution via a permutation procedure described by Stelzer et al. (2013). The analysis described above was repeated 100 times per participant, each time with a different random permutation applied to the rows of the target matrix. This yielded 100 correlation values for each patient, representing expected values from our workflow when no reliable relationship exists between neural data and target matrix (due to the permutation procedure). Then, a group-level empirical null distribution was estimated by randomly sampling one of the 100 performance metrics from each participant-level distribution and computing a permuted-group-average 10,000 times. This provided sufficient resolution at the group level at a fraction of the computational cost of fitting models to 10,000 permutations of the target matrix per participant for each test (i.e., each window, for each subset of items, in all analyses).

If m is the number of values in the permutation distribution and b is the number of values in the distribution larger than the true correlation value, then the one-tailed p-value can be computed (Edgington & Onghena, 2007; Phipson & Smyth, 2010) as:

$$p=\frac{b+1}{m+1}$$

Statistical significance was defined with respect to after adjusting the p-values to control the false discovery rate (FDR).

Finally, to understand the effects of regularization with structured sparsity (grOWL) versus more standard techniques, we compared models fit with grOWL regularization to those fit with L1 (LASSO) regularization.

### 4.2 Results

#### 4.2.1 Analysis 1: full window decoding with grOWL and LASSO

**Fig. 4.**
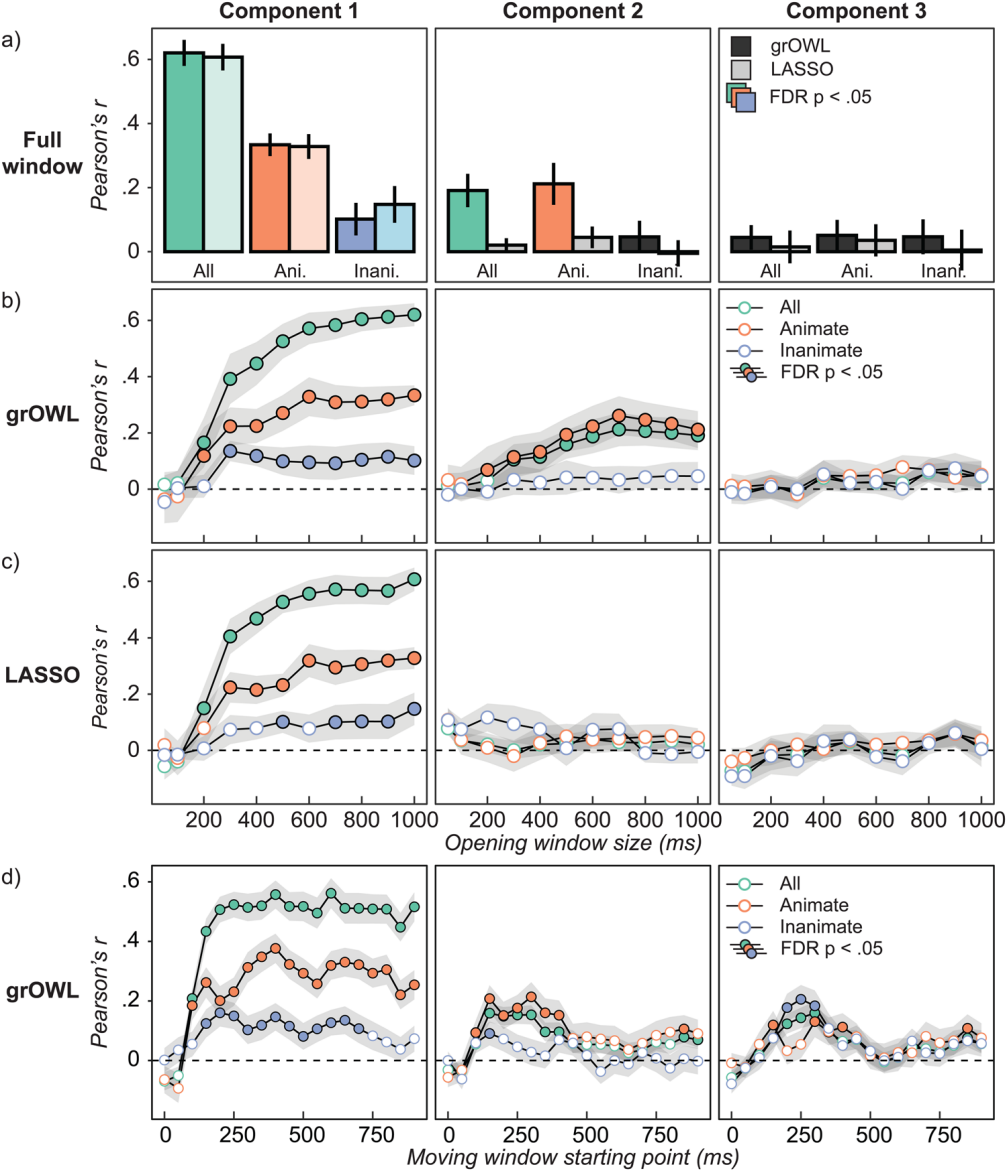
ECoG decoding results. Correlation between true and predicted coordinates along each latent dimension (columns) for models fit to all items (green), animate items only (orange), or inanimate items only (blue), and regularized with grOWL or LASSO. Error bars reflect standard error of the mean over participants. Each value is centered on the mean of its corresponding permutation distribution. For uncentered values, see Figure S4. Colored bars / filled circles indicate reliable decoding with FDR-corrected p < .05 for all points in a panel. (a) grOWL (darker bars) and LASSO (lighter bars) model performance when trained on the full 1000-ms trial epoch. (b) grOWL model performance within opening windows. (c) LASSO model performance within opening windows. (d) Analogous to (b) except that models are fit and evaluated within a 100 ms moving window instead of the opening window.

Figure 4a shows results from decoding the full 1000-ms time-window. For the first component, models fit with both grOWL and LASSO reliably decoded similarity structure across all items and for animate and inanimate subsets considered separately. Whereas this was the only structure discovered with LASSO regularization, grOWL regularization additionally showed reliable decoding of the second component across all items and within animates only. Thus, RSL with grOWL regularization revealed graded, multidimensional semantic structure in ECoG signals recorded from ventral ATL, while the contrasting pattern for LASSO suggests that additional constraints from grOWL aided in the discovery of multidimensional semantic structure.

#### 4.2.2 Analysis 2: opening window

To see how this structure emerges in the ECoG signal over time, we conducted an “opening window” analysis in which the same procedure was applied to an increasingly wide aperture of data, beginning with just the first 50 ms post-stimulus, extending to 100 ms, then growing by 100 ms up to 1000 ms. The opening window analysis evaluates when enough information has entered the spatiotemporal feature space to support decoding. The goal is not to localize a representation in time, but to identify when reliable decoding is first possible and when performance stops improving.

The results show reliable decoding of between-domain and within-animate structure along the first component by 200 ms, followed by within-inanimate structure by 300 ms (Fig. 4b). Decoding accuracy for superordinate and animate-subordinate structure continually improved with wider windows along component 1, but not inanimate-subordinate structure. Along the second component, reliable decoding was observed after 300 ms for between-domain structure and somewhat earlier (200 ms) for within-animate structure. Within-inanimate structure could not be reliably decoded along the second component at any window-size, nor could variation along the third component of the target matrix. Models fit with LASSO (Fig. 4c) also reliably decoded both within- and across-domain structure along the first component, beginning at 200 ms for cross-domain structure, 300 ms for a within-animate structure, and 500 ms for within-inanimate structure, but as with the full window, did not reliably decode the second (or third) component for any window size.

#### 4.2.3 Analysis 3: moving window

The opening window analysis indicates the latency with which the neural signal contains sufficient information for reliable decoding, but since each successive window contains all prior time points, it does not indicate whether/how the neural encoding of semantic information changes over time. Additionally, since larger windows contain more neural features, they afford a greater possibility of over-fitting training data. Consequently, the opening-window approach may fail to detect semantic information encoded only within a limited time-window. For these reasons, we fit models using the grOWL regularizer on a 100 ms *moving* window, beginning at 0 ms from stimulus onset and advancing in 50 ms increments. In this analysis, each window is the same size and thus contains the same amount of neural data. Otherwise, the analysis was identical to the opening-window variant.

Results are shown in Figure 4d. For component 1, reliable decoding was observed across domains and within each domain between 150–700 ms post-stimulus onset. Reliable decoding on components 2 and 3 was observed within a more limited time range. For component 2 (which best-separates the animate items), cross-domain and within-animate structure was reliably and equally-well decoded for windows beginning at 150–400 ms. For component 3 (which best separates inanimate items), cross-domain and within-inanimate structure was reliably and equally-well decoded for windows beginning at 200–300 ms. Together, the analyses suggest that, from around 200–400 ms post-stimulus, neural states measured by ECoG express both within- and between-domain semantic structure, for both animate and inanimate items, across all three components of the target matrix. That is, they express a graded multidimensional and domain-general semantic space.

#### 4.2.4 Analysis 4: moving-window reconstruction of full target similarity matrix

The preceding analyses consider decoding of each matrix component separately, allowing us to draw conclusions about which aspects of semantic structure are represented in the neural signal at which time points. The independent consideration of different dimensions, however, makes it difficult to compare results of RSL to RSA, since RSA considers correlations between full pairwise similarity matrices (the NSM and the RSM). To facilitate the comparison, we used the decoding models fit in Analysis 3 to construct a *predicted semantic similarity matrix* at each time-window*.* For each fitted model, we predicted coordinates of the corresponding held-out items along all three target matrix components, agglomerating these predictions across holdout sets to create a matrix of predicted coordinates for all items. Recall that the target coordinates are the first three singular vectors of the original semantic similarity matrix, weighted by the *square root* of the corresponding singular values (Fig. 3). Thus, to reconstruct predicted pairwise distances in the original target matrix, we need only take the product of the predicted-coordinates matrix with its transpose.

Each row of the resulting matrix contains predicted semantic similarities between an item and all other items. We then compared these predicted similarities to a target similarity matrix constructed directly from the three components of the original matrix that account for 90% of its variance. That is, for each item in the dataset, we computed the correlation between the predicted and true similarities to (a) all other items, (b) other items in the same domain, and (c) other items in the contrasting domain. For each metric, we then averaged the correlations across (1) all items, (2) just the animate items, or (3) just the inanimate items. These conditions thus allowed us to assess how well the decoders model semantic similarities both within and across each domain. The full procedure was carried out independently for each participant at each time-window. The results are essentially identical if the predicted similarities are instead correlated with the cosine similarity matrix describing the relationships among the Dilkina & Lambon Ralph (2013) feature vectors (Supplement E).

**Fig. 5.**
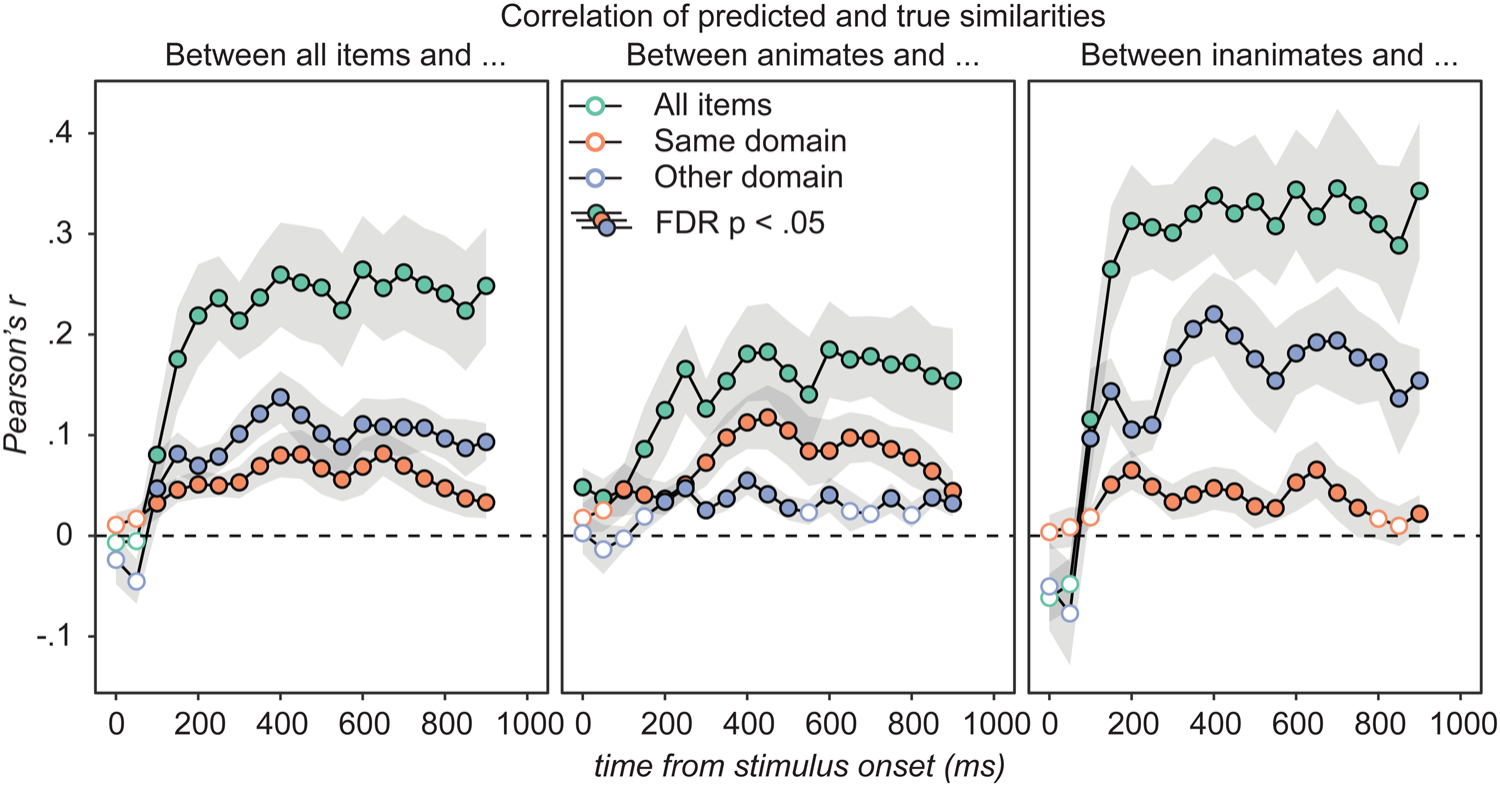
Correlation between predicted semantic similarities and the best-possible similarities reconstructed from the first three singular vectors/values of the true matrix. Error bars reflect standard error of the mean over participants. Decoding models fit with RSL reliably predict semantic similarities both within (orange dots) and between (blue dots) conceptual domains, for both animate (middle panel) and inanimate (right panel) stimuli, from about 100–200 ms post-stimulus onward. Correlation coefficients in each condition were then averaged across participants at each time-window.

The results are shown in Figure 5. Filled circles indicate where predicted/true correlations are reliably non-zero relative to a permutation-based null distribution with FDR of p < .05 (Stelzer et al., 2013). Reliable correlations were observed from 100–150 ms post-stimulus onward, whether computed across all items, within domain only, or between-domain only, and considering the full complement of items (left panel), animate items only (middle), or inanimates only (right).

**Fig. 6.**
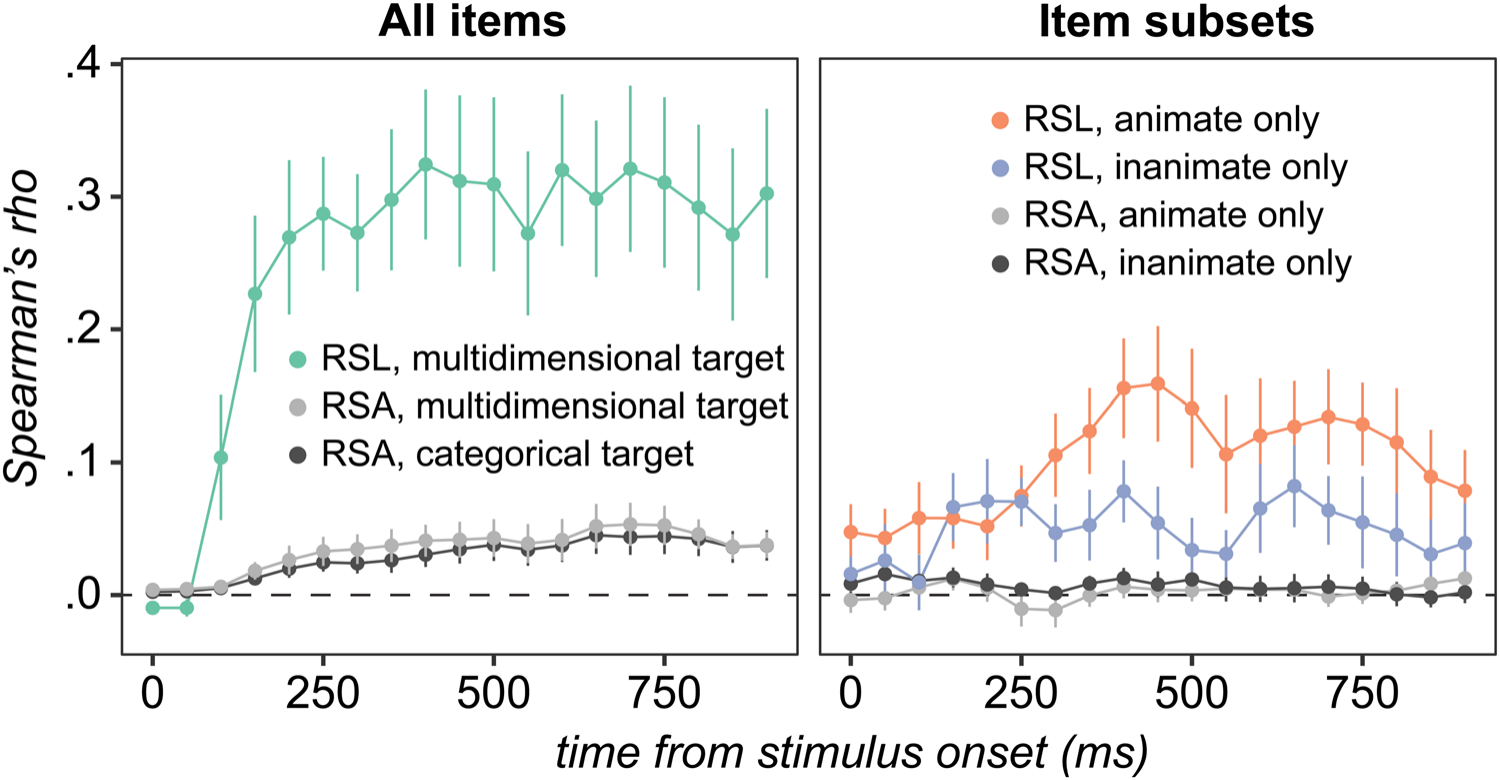
Comparing RSA and RSL on the same data. All correlations (Spearman’s rho) are averages over the 10 participants and error bars depict standard error. The left panel shows the correlation between the full predicted similarity matrix obtained using RSL and the multidimensional target RSM (green), as well as results obtained by replicating the RSA conducted by Chen, Shimotake, et al. (2016) on the same data used for RSL using the multidimensional (light grey) or a categorical (dark grey) target RSM. Differences between categorical and multidimensional RSA are insignificant, while differences between RSL and RSA are large and significant from 150 ms onward. The RSA results in the right panel are analogous to the left panel except that subsets of items are studied separately. RSA does not detect within-category structure while RSL does.

For comparison, we replicated the RSA analysis of Chen, Shimotake, et al. (2016) on the same data used for RSL and extended it to examine structure within animate and inanimate item subsets. When conducting RSA over all items, NSMs were correlated with both a multidimensional target RSM and a unidimensional categorical (i.e., binary) target RSM. Figure 6 (left) plots the average Spearman’s rho over participants within the same ROI reported by Chen, Shimotake, et al. (2016) for each temporal window and each target RSM in light and dark grey. The correlations with each target RSM are very similar, consistent with the original finding that RSA did not provide clear evidence about the kind of semantic information present in the vATL. The right panel shows results of RSA applied to animate (light grey) and inanimate (dark grey) item subsets. These correlations were indistinguishable from zero in every window.

Colored lines in the two panels show the correlations between the RSL predicted similarity matrix and the target matrix across all items (left panel) or within animates (right panel orange) and within inanimates (right panel blue). While reliably positive correlations over the full set of items were observed with both RSA and RSL from 150 ms onward (one-sample two-tailed t-tests, $\mu_{0}=0$, α=.05, all t(9) > 2.75), the correlation coefficients obtained by RSL were significantly larger in all windows with reliable effects (paired two-tailed t-tests, α=.05, all t(9) > 3.5). However, this is not a difference that requires a statistical analysis to appreciate: over windows with reliably positive correlations, the correlation coefficient obtained with RSL was between 6 to 13 times larger. A 10-fold increase in Spearman’s rho is a 100-fold increase in the proportion of variance explained. RSL produces a qualitatively different result from RSA, in terms of its sensitivity to detecting structure and its ability to determine what structure is present in neural signals.

### 4.3 Discussion

Replicating Chen, Shimotake, et al. (2016), if one only relied on RSA analysis then it would be easy to conclude that ECoG activity in vATL only encodes a discrete, binary distinction between animate and inanimate items, with no information about semantic structure within either domain considered independently. In stark contrast, the RSL analysis shows that ECoG signals measured in human vATL encode information about semantic similarity structure that is multidimensional (reliable decoding along three orthogonal components of the target matrix), graded (reliable decoding of varying degrees of similarity both within and between domains), and domain-general (reliable decoding of within-domain similarities for both animate and inanimate items). These properties are consistent with the predictions of the “hub and spokes” theory of semantic representation in the brain, which proposes that neural activity in vATL encodes a graded, multidimensional, and domain-general semantic vector space. It is also consistent with the converging sources of evidence that gave rise to that view, including patient studies (Patterson et al., 2006), neural stimulation (Pobric et al., 2007), and computational modeling (Rogers et al., 2004). In this sense, the RSL result resolves a seeming discrepancy between RSA findings and the broader literature.

## 5 General Discussion

We introduced this paper with a puzzle: neuropsychology, clinical neurophysiology, TMS, and computational modeling all suggest that the vATLs encode a semantic vector space of graded, multidimensional, and domain-general conceptual similarity structure, but direct tests of this hypothesis using representational similarity analysis have often yielded null results in vATL and positive results in brain areas not otherwise thought to encode semantic representations. In simulation, we showed that counter-intuitive limitations of RSA can obscure inferences about neurocognitive representation. When used to decode real semantic structure as measured by feature norms, RSA can produce positive results even if the underlying neural code is discrete and unidimensional, or null results even if the neural code does capture latent structure in the target matrix beyond the first component. Because RSA relies on an inherently unidimensional measure of association (i.e., correlation), it cannot reveal whether neural signals encode multidimensional structure. Because the technique does not fit any parameters to data, it requires the researcher to select features a priori when constructing the NSM (for instance, via ROI or searchlight analyses; see Frisby et al., 2023), and cannot learn to ignore irrelevant features among those selected. The simulations suggest that these characteristics of RSA may have contributed to the puzzling state of the literature—for instance, by yielding results suggesting that vATL only coarsely discriminates living from nonliving things (Clarke & Tyler, 2014; Chen, Shimotake, et al., 2016).

RSL addresses these limitations by using regression to predict coordinates of stimuli along the latent orthogonal dimensions of the RSM. In simulation, we showed how this approach can uncover multidimensional representational structure (by showing reliable conjoint decoding of two or more orthogonal dimensions of a target matrix) as well as graded structure (by showing reliable decoding both within and between semantic domains). The approach can also “select out” signal-carrying features from among those included as predictors in the model, and so can be applied to all potentially signal-carrying neural features at once, without requiring the theorist to pre-select an ROI or to look only within small, independent searchlights (see Cox & Rogers, 2021; Frisby et al., 2023).

Applying the approach to large neural datasets requires model regularization. We illustrated how hypothesized patterns of structured sparsity in the neural signal can constrain model fit via grOWL regularization and applied this approach to discover semantic structure in an ECoG dataset where prior work using RSA found only a binary animacy code (Chen, Shimotake, et al., 2016). We replicated this analysis using the same RSA approach, and further showed that RSA yields null results when assessing whether the ECoG signals express within-domain semantic structure. In contrast, RSL with grOWL regularization uncovered a graded and multidimensional semantic space capturing similarities within and between both animate and inanimate domains—consistent with conclusions drawn about the nature of semantic representations in the ATL hub from other cognitive and clinical neuroscience sources. These results thus suggest that discrepancies in the literature between studies employing RSA versus other sources of evidence may reflect limitations of the RSA approach as typically practiced.

### 5.1 Validity of grOWL assumptions about neural signal

The RSL models we have deployed were fit with a regularization function designed to promote discovery of a *row-sparse* decoding matrix (the grOWL loss). We hypothesize that such structure reflects three characteristics of neuro-semantic representation specifically, and neuro-cognitive codes generally. First, the signal is likely to be sparse: of all neural measurements taken in a given experiment, only a relatively small proportion is likely to encode the target information of interest. The sparsity assumption serves a useful role in decoding because it pressures many coefficients in the regression model to zero, indicating that the corresponding features are not useful in decoding the target information. In this sense, sparsity automatically serves the function of feature-selection that, in RSA, must be handled a priori based on an ROI, searchlight, or other method (Cox & Rogers, 2021). We hypothesize neural codes to be relatively sparse in general simply because brains support many different cognitive, perceptual, motor, language, and affective functions—consequently, the likelihood that a given neural population is important for the specific function targeted by the investigator is relatively small.

Second, we hypothesize that the signal is *redundant*: it is unlikely that a given target structure is only encoded by a single voxel, or a single timepoint at a single electrode, etc. Any cognitive construct of interest—feature, category, or dimension in a representational space—is unlikely to be encoded by the activation of a single local neural population, such as a single voxel. More likely, such information involves multiple neural populations, in which case those populations that *do* encode target information will exhibit some degree of correlation with one another. This assumption is captured by the grOWL loss because it encourages solutions in which intercorrelated sets of neural features that help to predict target structure receive similar, non-zero coefficients in the decoder.

Finally, we hypothesize that the neural code is unlikely to be axis-aligned with the dimensions of the target representation space. That is, a given neural feature is unlikely to encode variation along just one dimension of a multidimensional target space without also encoding some information about variance along other dimensions. In grOWL, this hypothesis is expressed as a preference for learning a row-sparse decoding matrix—coefficients on a given neural feature should either be all zero (the feature is unimportant) or all non-zero (the feature explains some variance along each dimension). The reason is simply that, for any target vector space, there exists only a small and finite number of axis-aligned encodings, but an infinite number of non-axis-aligned encodings. Aligned and unaligned encodings express the same information about similarities among objects of representation; so, absent some explicit pressure for brains to learn axis-aligned representations, it is unlikely that an axis-aligned encoding will occur by chance. Additionally, ECoG electrodes are influenced by voltages generated by a mix of individual local neurons—so even if individual neurons are selectively tuned to axis-aligned dimensions, the net responses recorded at the electrode are likely to reflect a blend of these dimensions.

While the grOWL regularizer generally prefers decoding models with these properties, note that the relative strength of these constraints is determined by hyperparameters that can be tuned via cross-validation to fit the data. If the best solution is not particularly sparse, the tuning process will select a small weight for the sparsity term in the optimization, leading to a solution in which many features are chosen. If the signal-carrying features are not particularly correlated, the tuning process can select hyperparameters that relax the grouping of features into sets that share the same weights. Thus, grOWL regularization is quite flexible with respect to how rigidly the various constraints are enforced.

The current study suggests that the grOWL assumptions are useful for understanding information encoded in ECoG voltages: when decoding the full time-window, regularization with grOWL revealed multidimensional similarity structure, whereas decoding with the sparsity assumption only (LASSO) found only unidimensional structure. We emphasize, however, that the RSL framework can be deployed with any form of model regularization. Alternative hypotheses about the likely structure of neural encodings can be formulated as different regularization costs, and the decoding success of models fit with different regularizations can then be compared to empirically evaluate the different assumptions. Prior computational work has contrasted grOWL regularization to other approaches (see Oswal et al., 2016 for comparisons to some other related methods); we hope the current results with grOWL will inspire other scientists to experiment with alternative losses to better understand the nature of neuro-cognitive codes.

### 5.2 Implications for alternative theories of ATL function

The current results challenge an alternative proposal about the role of ATL in semantic cognition, namely that they support knowledge of some conceptual categories and not others (Mahon & Caramazza, 2011; Malone et al., 2016; Simmons et al., 2009). Such a view is consistent with prior imaging work (e.g., Anzellotti et al., 2011), including the multivariate decoding studies cited earlier (Chen, Shimotake, et al., 2016; Clarke & Tyler, 2014), suggesting that ATL activations differentiate animates from inanimates but do not otherwise encode or differentiate finer categories. This alternative perspective has been difficult to reconcile with neuropsychological evidence, showing that anterior temporal atrophy and hypometabolism in semantic dementia degrades knowledge of animates and inanimates equally (Lambon Ralph et al., 2007; Noppeney et al., 2007), and with TMS and direct grid stimulation evidence showing that stimulation of both left and right ATL reliably slows semantic processing for both animates and artefacts (Pobric et al., 2010; Shimotake et al., 2015). It also struggles to explain the sensitivity of ATL-related semantic impairment to continuous and graded semantic structure of test stimuli for both animates and inanimates (Patterson et al., 2006; Rogers et al., 2006). The current results suggest that prior work may have failed to discover graded cross-domain semantic structure in ATL responses, not because such structure is absent from the measured responses, but because limitations in both fMRI and analytic techniques make such structure difficult to detect.

It is worth noting that intra-domain similarities were more robustly decoded for animate compared to inanimate items. Considering each dimension separately in the opening window analysis, within-inanimate decoding was only reliable for the first latent dimension. In the moving window analysis, where models were trained on 100 ms increments of data without the full history of activation from stimulus onset, within-inanimate decoding was reliable for components 1 and 3, but transiently. These are the components that best differentiate inanimates in the target matrix (see Fig. 1). When predicting pairwise distances within moving windows, correlations with true similarities were reliable but the smallest for within-inanimate structure (i.e., the “other domain” correlations in the middle panel of Figure 5, and “same domain” correlations in the rightmost panel).

We attribute this general pattern to differences, not in the neural code itself, but in the target similarity structure of animate and inanimate concepts. Semantic subcategories of inanimate objects are only weakly differentiated, not just in the current dataset, but in norming studies more generally and in other approaches to characterizing semantic structure (e.g., Devlin et al., 1998; Garrard et al., 2001; McRae et al., 1997; Tyler et al., 2000). Indeed, this difference in statistical structure is precisely what leads, under some theories, to apparent category-specific patterns of semantic impairment (Devlin et al., 2002; Lambon Ralph et al., 2007) and functional activation (Rogers et al., 2006). The differences can be seen in Figure 1, where animate items fall into somewhat well-differentiated subcategories in components 1 and 2 while inanimates are uniformly distributed with poorly differentiated subclusters even along component 3. Accordingly, pairwise similarities reconstructed from the first three singular vectors of the full semantic matrix are more accurate for animate than inanimate items (see Supplement D). Thus, the structure of the semantic matrix itself *requires* that decoding be worse within inanimates than within animates, precisely because inanimates are less well structured. Yet despite this intrinsic disadvantage, within-inanimate similarities were still reliably decoded along multiple components of the embedding and in the reconstructed similarity matrix, illustrating that the neural signal in vATL does express intra-domain semantic structure even for inanimate stimuli.

In summary, the results suggest the vATLs encode a multidimensional representation space that captures the conceptual similarities existing among a variety of different concepts, including both animate and inanimate items (Lambon Ralph et al., 2007; Patterson et al., 2007; Rogers & McClelland, 2004). Better decoding within animates may reflect the fact that animate subcategories are better-differentiated, so that intra-domain similarities are better-approximated by a low-rank decomposition.

We also note that the current results pertain only to the nature of the semantic information encoded within the span of vATL where electrodes were situated in our patient sample. They do not bear on claims of possible category-specific representation in other parts of the cortex—for instance, that the left infero-parietal cortex and posterior medial temporal gyrus play a special role in knowledge about tools (Chen, Garcea, et al., 2016; Garcea et al., 2018; Ishibashi et al., 2016; Kalénine & Buxbaum, 2016) or that earlier visual areas are dedicated to representing different object categories (Cichy et al., 2014; Connolly et al., 2012; Downing et al., 2001; Kanwisher et al., 1997; Kriegeskorte, Mur, Ruff, et al., 2008; Mahon et al., 2009; Sha et al., 2015). Indeed, prior work from (Chen et al., 2017) showed how domain-general semantic representations can arise in vATL even as graded category-specificity emerges in other parts of the cortical semantic network, based on empirically measured patterns of white-matter connectivity across core areas. Evaluating claims of category-specificity in future work may benefit from adopting the RSL approach developed here.

### 5.3 Implications for the broader literature

The characteristics of RSA we have identified carry additional implications for interpretation of the broader literature. A recent review (Frisby et al., 2023) identified 24 studies that have applied RSA to the discovery of semantic representations in the brain, with positive results observed across multiple cortical regions, including the posterior temporal cortex (Connolly et al., 2012), angular gyrus (Fairhall & Caramazza, 2013; Fernandino et al., 2022), left perisylvian cortex (Devereux et al., 2013), posterior cingulate (Fairhall & Caramazza, 2013), and prefrontal cortex (Carota et al., 2017). Simulation 1 suggests, however, that RSA will yield a positive result for any property correlated with the animate/inanimate distinction, including even discrete binary properties. Many features are confounded with animacy: inanimate items tend to be more familiar, less visually complex, more associated with action plans, less associated with motion, more likely to have lines and corners, less predictable from color or texture, etc. (see Chen & Rogers, 2014 for a review). Most RSA papers do not report the magnitude of correlation between RSM and NSM where results are significant, instead focusing on whether the estimated correlation coefficient is reliably non-zero across participants. As shown by the prior RSA analysis of the same ECoG data explored here (Chen, Shimotake, et al., 2016; gray line in Fig. 5), this can happen even when mean correlations are very small. Moreover, several studies use target RSMs with only a small number of rows/columns—sometimes as few as five (Connolly et al., 2012; Fairhall & Caramazza, 2013), meaning that correlations are computed across just 10 cells of the matrix (i.e., the lower triangle of a 5 x 5 RSM). Such small numbers increase the likelihood that a small-but-non-zero correlation is driven by some arbitrary property of the chosen stimuli or categories. Together these observations raise the possibility that the literature contains misleading positive results—brain areas whose responses encode unidimensional characteristics weakly confounded with animacy, rather than multidimensional semantic structure. Testing this possibility for various brain areas hypothesized to encode semantic structure will require analyses like those we have developed here.

## 6 Conclusion

In cognitive science, semantic representations are often construed as vector spaces that encoded graded, multidimensional similarity structure among the concepts experienced in our verbal and nonverbal world. Through application of a new technique for mapping representational similarity in neural activity, we have shown that neural signals in vATL encode such a space. In so doing, we have identified some limitations of representational similarity analysis, a widespread technique commonly thought to reveal graded and multidimensional representational structure. The work resolves an important discrepancy between behavioral and neuroimaging results in prior work and suggests a new approach to discovering representational structure in neural data more generally.

## Supplementary Material

Supplementary Material

## Data Availability

RSL models were fit using the WISC MVPA toolbox (https://doi.org/10.5281/zenodo.6975270). Analysis of RSL models was conducted using custom MATLAB code (https://doi.org/10.5281/zenodo.7566607), and the data and script necessary to replicate them are available via the Open Science Framework (https://osf.io/axseq/). R scripts for generating figures based on the correlation analyses are on GitHub (https://doi.org/10.5281/zenodo.7566609).
