# Supplementary material for: Representational similarity learning reveals a graded multidimensional semantic space in the human anterior temporal cortex
Source: Imaging Neurosci (Camb). 2024 Feb 22;2:imag-2-00093. doi: 10.1162/imag_a_00093 (PMC12224414; doi:10.1162/imag_a_00093)
Supplement: Supplementary Material [file imag_a_00093-supp.pdf]

Supplemental materials for: Representational similarity learning reveals a graded multidimensional semantic space in the human anterior temporal cortex

Christopher R. Cox[1], Timothy T. Rogers[2], Akihiro Shimotake[3*], Takayuki Kikuchi[4], Takeharu Kunieda[4,5], Susumu Miyamoto[4], Ryosuke Takahashi[3], Riki Matsumoto[7], Akio Ikeda[6,] Matthew A. Lambon Ralph[8]

Affiliations:
1. Dept. of Psychology, Louisiana State University, Audubon Hall, Baton Rouge, LA 70803
2. Dept. of Psychology, University of Wisconsin, Brogden Hall, Madison, WI 53706
3. Dept. of Neurology. Kyoto University Graduate School of Medicine, Kawaharacho, Shogoin, Sakyo-ku, Kyoto, 606-8507, Japan
4. Dept. of Neurosurgery, Kyoto University Graduate School of Medicine, Kyoto, Japan
5. Dept. of Neurosurgery, Ehime University Graduate School of Medicine, Shizukawa Toon city, Ehime, 791-0295, Japan
6. Dept. of Epilepsy, Movement Disorders and Physiology, Kyoto University Graduate School of Medicine, Kyoto, Japan
7. Div. of Neurology, Kobe University Graduate School of Medicine, Kusunoki-cho, Chuo-ku, Kobe, 650-0017, Japan
8. MRC Cognition and Brain Sciences Unit, 15 Chaucer Rd., Cambridge, UK, CB2-7EF

Open access: For the purpose of open access, the UKRI-funded author MALR has applied a Creative Commons Attribution (CC BY) license to any Author Accepted Manuscript version arising from this submission.

Contents

Supplement A: Categorized experimental stimuli 2
Supplement B: Formal description of the RSL approach. 3
Supplement C: RSL and negative correlations 6
Supplement D: Reconstructing item similarity from SVD coordinates 10
Supplement E: Correlation between predicted and full-rank semantic distances 13
Supplement F: Temporal generalization of models fit within moving windows 14
Supplement G: Anatomical distribution of decoding model weights across vATL 16
Supplement H: Predicted embeddings 19
Supplemental References 21

Supplement A: Categorized experimental stimuli

Table S1. Categorized experimental stimuli

Domain	Category	Words
Animate	*Bird*	duck, eagle, ostrich, owl, peacock, penguin, rooster, swan
	Bug	ant, bee, butterfly, caterpillar, fly, ladybird, snail, spider
	Land	bat, bear, camel, cat, cow, deer, dog, dragon, elephant, fox, frog, giraffe, goat, gorilla, horse, kangaroo, lion, monkey, mouse, pig, rabbit, raccoon, rhinoceros, sheep, snake, squirrel, tiger, tortoise, zebra
	Water	crab, fish, lobster, seahorse, whale
Inanimate	*Clothes*	dress, glove, mitten, skirt
	Music	bell, cymbals, drum, flute, guitar, harp, piano, trumpet, violin, whistle
	Object	anchor, barrel, basket, cake, cannon, chain, church, cigar, clock, crown, fence, gun, hammer, iron, kite, ladder, nut, pram, shell, slide, snowman, suitcase, swing, toaster, vase, web, wheel, windmill, yoyo
	Vehicle	aeroplane, helicopter, motorcycle, ski, sled, tractor, train

Supplement B: Formal description of the RSL approach.

Let $\boldsymbol{S}$ be a symmetric $n \times n$ target matrix of approximate rank r describing pairwise cognitive similarities amongst *n* items, which is decomposed via singular-value decomposition (SVD) into an $n \times r$ matrix $\boldsymbol{U}$ of r orthonormal singular vectors and an $r \times r$ diagonal matrix $\boldsymbol{D}$ containing the corresponding r singular values (**Figure 2A**). The matrix product $\boldsymbol{U}\sqrt{\boldsymbol{D}}$ then contains root-weighted singular vectors that can be viewed as encoding the coordinates of each stimulus within an r-dimensional latent semantic space, while the product of this matrix with its transpose provides a rank r approximation of the original similarity matrix. That is:

$$S_{n\times n} \cong U_{n\times r} D_{r\times r} U^T = U\sqrt{D}\,(U\sqrt{D})^T$$

Let $\boldsymbol{X}_{n\times m}$ be a data matrix recording neural responses to each of n stimuli over m neural features (e.g., voxels in fMRI, electrode voltages at each timepoint in ECoG, etc.). To assess whether some weighted combination of these features jointly encode the target similarities, we seek a *decoding matrix* $\boldsymbol{\beta}_{m\times r}$ such that the matrix product $\boldsymbol{X\beta}$ produces *r* coordinates for each item, deviating as little as possible from those in target matrix $\boldsymbol{U}\sqrt{\boldsymbol{D}}$. This search can be formulated as the convex optimization:

$$argmin_{\beta}\left\|U\sqrt{D} - X\beta\right\|_F$$

That is, we seek $\boldsymbol{\beta}$ values that minimize the Froebenius norm (i.e., sum of squared differences) between the true root-weighted singular vectors $\boldsymbol{U}\sqrt{\boldsymbol{D}}$ and the predicted values $\boldsymbol{X\beta}$. The optimization essentially computes a simultaneous regression predicting all *r* coordinates for each stimulus from weighted combinations of the neural responses they evoke. Each column of the resulting decoding matrix contains one

coefficient for each neural feature and predicts an item's coordinate along one component of the target matrix $\boldsymbol{U}\sqrt{\boldsymbol{D}}$ (ie, one dimension of the semantic space).

As noted earlier, there are typically many more neural measurements (predictors) than stimuli (i.e., target data points)—that is, $m \gg n$. Thus, the optimization just defined is under-constrained and must be regularized with an additional loss H, itself a function of the coefficient matrix $\boldsymbol{\beta}$:

$$argmin_{\beta}\left(\left\|U\sqrt{D} - X\beta\right\|_F + \alpha H(\beta)\right)$$

…which is also convex so that a weighted sum of this function and the original reconstruction loss together yield a single unique solution that can be discovered via gradient descent. The constant α is a free weighting parameter governing the importance of the regularization loss relative to the model error.

For RSL we wish to use a regularization function that promotes solutions respecting the sparsity, redundancy, and spanning assumptions described earlier. Oswal et al. (2016) formulated a regularizer that accomplishes these objectives by constraining the structure of the decoding matrix $\boldsymbol{\beta}$ in a single convex loss called the *group ordered weighted LASSO* or *grOWL.* The approach generalizes both the group LASSO (Yuan & Lin, 2006) and the ordered-weighted LASSO (Figueiredo & Nowak, 2016) to favor solutions that are (1) row-sparse (rows of $\boldsymbol{\beta}$ are either all zero or all non-zero), implementing both the sparsity and spanning constraints, but (2) with subgroups of selected features sharing similar coefficients if their activations covary strongly together (implementing the redundancy constraint). The grOWL loss is specified as:

$$H(\beta) = \sum_{i=1}^{m} w_i \left\|\beta_{[i]\cdot}\right\|_2$$

Here m is the number of neural features (rows of $\boldsymbol{\beta}$), $\boldsymbol{\beta}_{[i]\cdot}$ is the row of $\boldsymbol{\beta}$ with the i-th largest 2-norm (i.e., the ith longest coefficient vector), and w_i is a vector of non-negative, non-increasing weights (for instance a vector of positive values that decay linearly from an initial constant). Intuitively the loss works as follows. The first element of the weighting vector w is stipulated to be as large or larger than all other elements, and this weight is applied to whichever neural feature has the largest coefficients in the decoding matrix (i.e., the 2-norm or length of the corresponding row). Thus, the feature with the largest coefficients exerts the largest regularization cost. The optimization must therefore put the largest weights on the most informative feature to make it “worth” the high regularization cost. If the most informative feature is also correlated with many other features, their total cost can be further minimized by placing similar weights across all such features, since the cost for each such weight declines with the weight sequence in *w*. Thus, grOWL spontaneously clusters correlated signal-carrying neural features and gives these a similar weight, effectively averaging them. Finally, the weighting vector ensures that coefficients on features that do not reduce error beyond those already selected will be pressured to zero, ensuring overall sparsity. Formal analysis of the grOWL regularizer and mathematical proofs of its row-sparse and clustering properties were provided by Oswal et al. (2016).

Supplement C: RSL and negative correlations

The analysis protocol presented in this paper involves generating out-of-sample predictions with k-fold cross validation and then aggregating those test-set predictions into a single matrix before correlating with the target embedding all in one go. This is in contrast with a more conventional protocol in which the performance metric is computed separately within each fold and then averaged. When the metric is something like mean squared error or classification accuracy, the two protocols are equivalent. With correlation, however, the two protocols yield different values.

Pearson's r compares variables with zero mean and unit variance. Consequently, when correlations between target and predicted embeddings are computed separately by holdout-set, the scale of the predictions relative to the target is disregarded. By aggregating over test sets and correlating one time, the standardization is done at the level of the full dataset and not for individual test sets. We feel this is a better metric of whether our models can embed all items appropriately within the target similarity space—it matters how items are situated with respect to one another even if they are in different test sets.

When models are fit with cross validation, the targets (and the data) are standardized with respect to the values in the training set. Thus, the model predictions, including test set predictions, are on the scale of the standardized *training set*. This means that, to aggregate over test sets and correlate with the full set of targets, the mean and variance of the target embeddings in the *training set* need to be added back into the predictions for the corresponding *test set.*

Applying this adjustment will introduce negative correlations between target and predicted embeddings for models that have been regularized to have weights that are all zero. This will happen when relationships between the neural data and the target embedding is weak or non-existent, because putting a weight on any neural feature increases the model cost function without reducing the prediction error on the training set. In such cases, the unadjusted model predictions will all be zero, and the adjusted predictions will be the mean of the training set. If the mean of the training set is greater than the grand mean of the full set, then the mean of the test set must be less than the grand mean of the full set. Thus, adjusted and aggregated test set predictions will be negatively correlated with the full target embedding when the model has all zero (or trivially small) weights due to the LASSO or grOWL regularization penalty. These concepts are illustrated in Figure S1 in an imagined case where all-zero models are learned on each of three cross-validation folds.

What we found in practice is that grOWL is "more aggressive" about assigning zero or trivially small weights than LASSO in the earliest and smallest time-windows. We consider this to be desirable behavior because we do not expect much semantic activity in the ATLs at 50ms post stimulus onset. Figure S2 is analogous to Figure 4 in the main paper, except that the correlation values are not centered on the mean of their corresponding permutation distribution. This reveals the negative correlations between grOWL model predictions and the target embedding for small windows where we do not expect semantic activation to be reliably present, and general consistency of performance elsewhere. These negative correlations are an expected artifact of our analysis protocol and do not change the interpretation of our results.

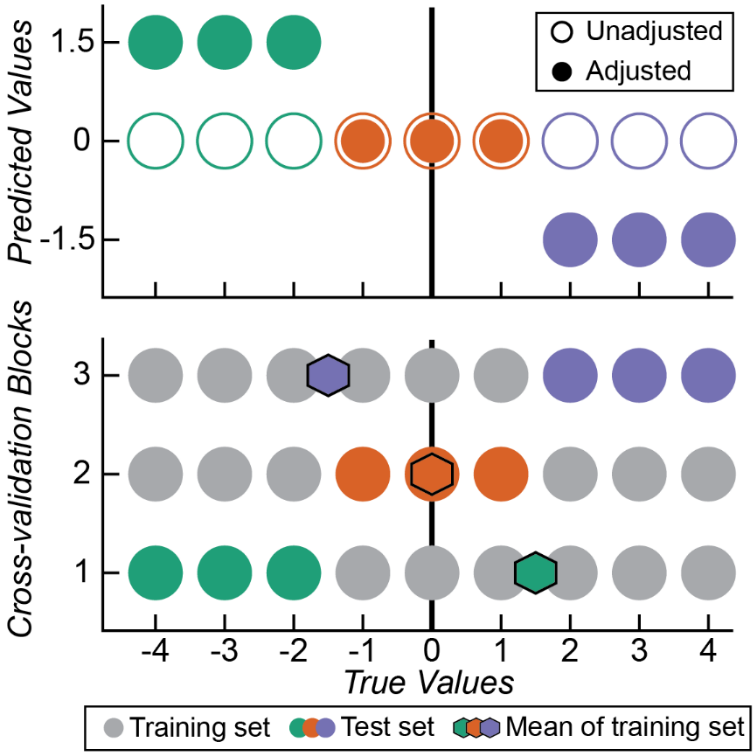

Figure S1 A schematic depiction of the relationship between test sets, training set means, the sample grand mean, and model predictions when the model consists of zero-weights on all neural features. A zero-weight model can occur when model regularization involves the LASSO penalty and the cost of applying a non-zero weight does not outweigh the benefit of reducing prediction error. If the mean of the test set differs from the grand mean, the mean of the test set must also differ from the grand mean. Importantly, if the mean of the test set is less than the grand mean, the mean of the training set must be higher. Unadjusted predictions (i.e., those that ignore the mean of the training set) will be all zero when the model weights are all zero; adjusted predictions are scaled by the training set mean. Leads to systematically negative correlations between true and predicted values for models regularized to have all-zero weights.

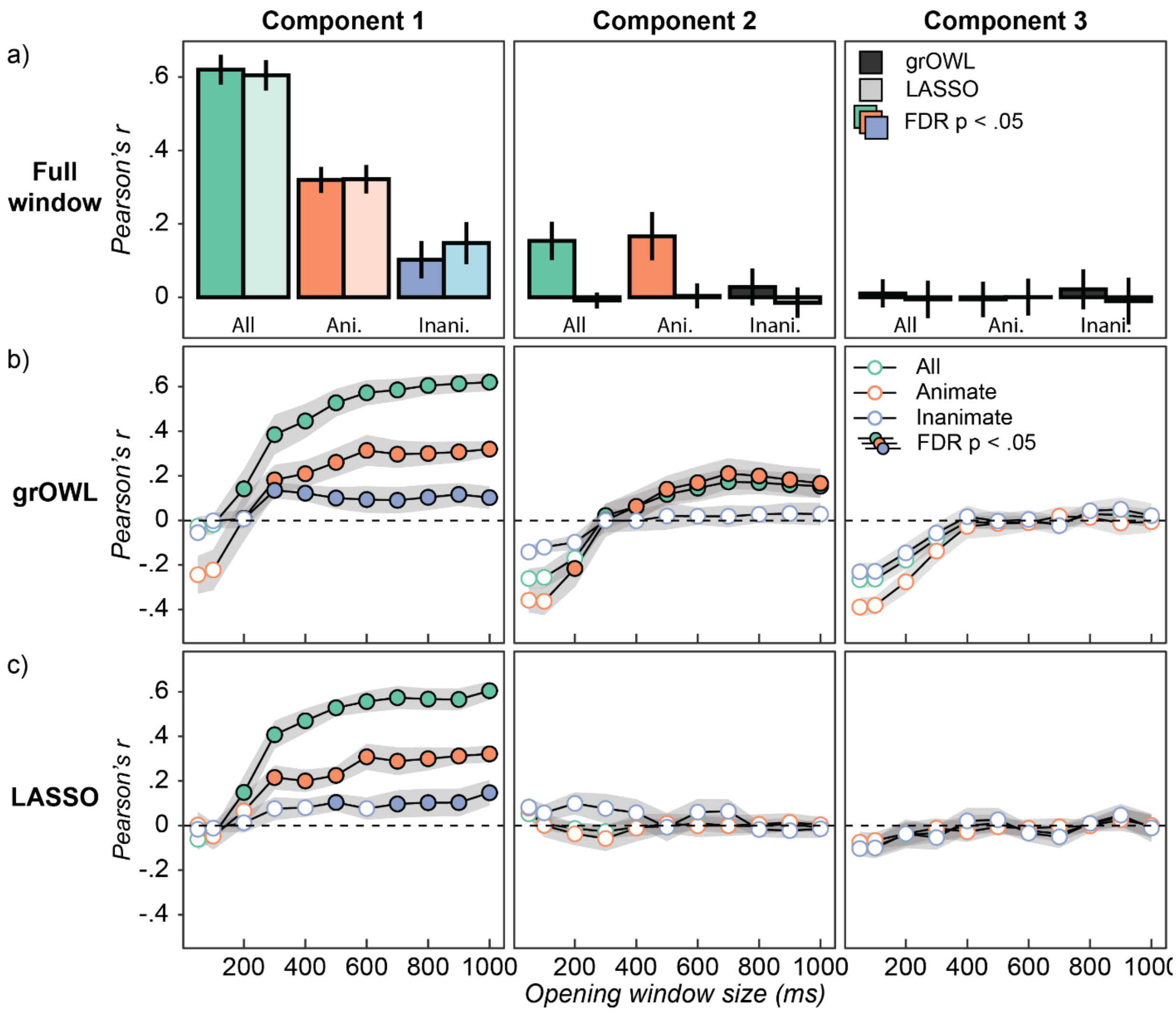

Figure S2. Uncentered ECoG decoding results. This figure is completely analogous to Figure 4 in the main paper, except that the raw correlations are presented, without centering on the mean of the permutation distribution. We see that the predictions of models fit with grOWL to small windows, including the least activation since stimulus onset, tend to be negatively correlated with the target values. This is due to the models being assigned very small weights, such that the variance in the predicted values associated with the training set means is large relative to the variance associated with the variability in the neural data. It is possible for negative correlations to be reliably higher than the expected value until the permutation-simulated null, as seen for the animate-subordinate structure along dimension 2 in the 200-ms window.

Supplement D: Reconstructing item similarity from SVD coordinates

The main analysis uses the first three root-weighted singular vectors as the target for fitting decoding models with RSL. Predicted coordinates are then used to generate predicted semantic distances between pairs of held-out stimuli. To understand the possible causes of domain differences in prediction accuracy, we considered how well pairwise distances can be reconstructed from the first three singular vectors and values computed from the true matrix with a subset of items excluded. The goal was to assess whether the first three components capture inter-items distances equally well for both domains or whether such distances are better approximated for one domain than another.

To this end we conducted the following analysis. We generated 10,000 random splits of the semantic norming data, each consisting of 90 training items and ten test items (five animate and five inanimate) as in the ECoG study. For each, we computed the SVD on the semantic distance matrix for only the items in the training set. We then projected test-set items into the resulting space by matrix multiplying each item's similarity with the training set items:

$$\widehat{\boldsymbol{C}}_{\boldsymbol{q}\times\boldsymbol{r}} = \boldsymbol{S}'_{\boldsymbol{q}\times\boldsymbol{p}}\boldsymbol{U}_{\boldsymbol{p}\times\boldsymbol{r}}\boldsymbol{D}_{\boldsymbol{r}\times\boldsymbol{r}}; \boldsymbol{S}' = \left(\boldsymbol{S}_{\boldsymbol{ij}}\right)_{\substack{i\in test \\ j\in training}}$$

Here $q = 10$ is the number of test set items, $p = 90$ is the number of training-set items, $r = 3$ is the rank of the embedding, $\widehat{\boldsymbol{C}}_{\boldsymbol{q}\times\boldsymbol{r}}$ is a matrix containing estimates coordinates for the test items, $\boldsymbol{S}'$ is a matrix containing the true distances from test items to training items, and $\boldsymbol{U}$ and $\boldsymbol{D}$ are matrices containing the singular vectors and values, respectively, computed the training items. From the estimates coordinates in $\widehat{\boldsymbol{C}}_{\boldsymbol{q}\times\boldsymbol{r}}$, the predicted similarity matrix can be obtained as $\widehat{\boldsymbol{S}}_{\boldsymbol{q}\times\boldsymbol{q}} = \widehat{\boldsymbol{C}}\boldsymbol{D}\widehat{\boldsymbol{C}}^{\boldsymbol{T}}$. To measure how well the

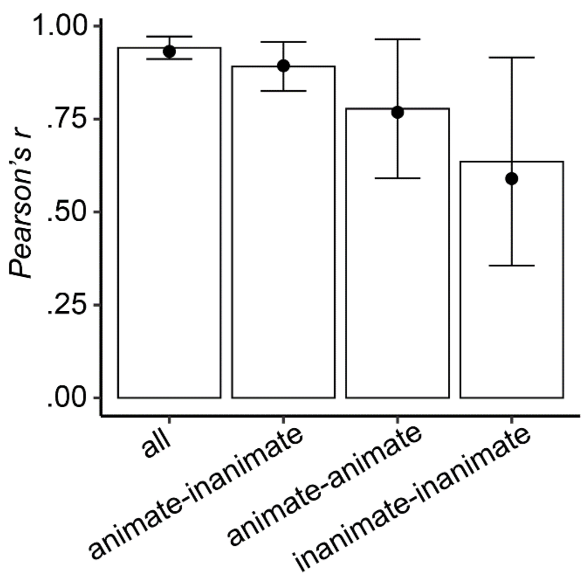

Figure S3. Correlation between true and reconstructed similarity matrices. Reconstructed similarity matrices are based on a SVD of a subset of the full similarity matrix expressing the pairwise similarities for 90 items, which is then used to reconstruct the similarities for the remaining 10 items as described in the text. This process was repeated 10,000 times with different 90/10 splits and each reconstruction was correlated with the corresponding true pairwise similarities. Bars show the average correlations over all similarities, just the between-category similarities (animate-inanimate), and just the within-category similarities (animate-animate and inanimate-inanimate). Error bars report the standard deviation of the means. The results for the specific 90/10 split used in the analysis of ECoG data reported in the main document are shown as black dots.

true distances are captures by the SVD, we computed correlations of lower-triangular values between $\boldsymbol{S}$ and $\widehat{\boldsymbol{S}}$, either over all values at once or correlating the animate-animate, inanimate-inanimate, and animate-inanimate similarity values separately.

Figure S3 shows the average correlations for each selection of similarities over the 10,000 splits as bars with error bars reflecting the standard deviation over splits. The mean over the 10 cross-validation splits used in the ECoG study are overlaid as points. Predicted similarities that include or isolate between-category structure are most correlated with the true similarity structure. Predicted within-category similarities are less correlated, with predictions for inanimate items being less accurate than for animate items. In other words, a low-rank approximation of the semantic similarity matrix best captures between-domain distances and provides a better approximation of

within-animate than within-inanimate semantic structure. Thus, decoding models face an intrinsic disadvantage at capturing within-domain structure generally, and within-inanimate structure specifically.

Supplement E: Correlation between predicted and full-rank semantic distances

In evaluating how well the RSL decoding models predict pairwise semantic similarities, the main paper reports the correlation between predicted similarities and similarities reconstructed directly from the three singular vectors computed from the "true" full-rank matrix. We will refer to this as the *low-rank* similarity matrix. The low-rank similarity matrix represents the best possible prediction, given that only three components of the true matrix were used to fit the decoding model. Here we report correlations with the *full-rank* similarity matrix—the actual pairwise similarities amongst feature vectors as described in the main text. The low-rank and full-rank similarity matrices are very similar, $r = .94$, such that the low-rank similarity matrix accounts for ~90% of the variance in the full-rank matrix. As one would expect given the high correlation, **Figure S4** and **Figure 5** from the main document show the same pattern of results. Filled circles indicate significance at Westfall-Young FWER corrected $\alpha = .05$.

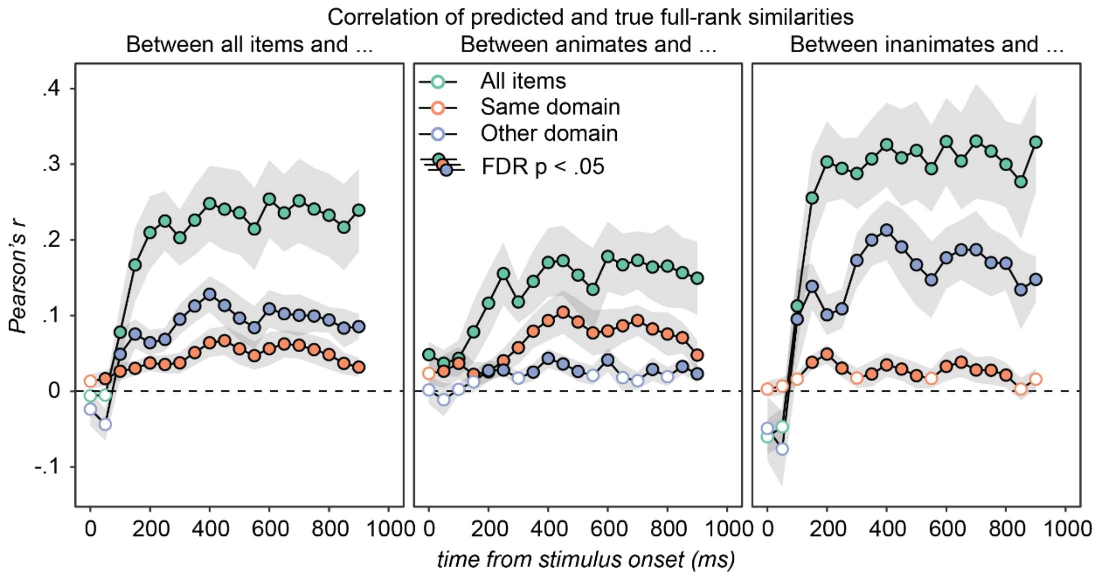

Figure S4 Correlation between predicted semantic similarities and *full-rank* cosine similarities as computed from the feature vectors. These results are very similar to **Figure 5** in the main document which involves the *low-rank* similarity matrix, as reconstructed from the three semantic dimensions modeled in the primary analysis. The results

are so similar because the first three components account for ~90% of the variance in the full-rank cosine similarity matrix. The interpretation is the same as **Figure 5**.

Supplement F: Temporal generalization of models fit within moving windows

The model fit within a moving window can be used to decode target signal from the data within all other moving windows, forming a temporal generalization profile forwards and backwards through time. In Figure S5, each row in each panel is the temporal generalization profile for one 100ms window. Colored points are significant with FDR-corrected $p<.05$, correcting over the three panels in a single row (i.e., the multiple subsets relative to a shared dependent variable). Profiles are shown for RSA-style paired-matrix correlations (Cosine Similarities) and for correlations between model predictions and the target embedding along each semantic component. Each analysis can be performed over all items or by restricting to within-animate or within-inanimate similarities.

Generalization is better at later time points, but some degree of reliable generalization is seen through time for most models that perform well on the window it was fit within for component 1 and the aggregate cosine similarities. However, this is not seen when decoding components 2 and 3. Models fit early in the epoch do not generalize to later time points, and models fit late in the epoch do not generalize to early in the epoch.

The terms “early” and “late” are intentionally vague. We are not arguing for a qualitative, discrete shift in representational content at a particular point in time. If concepts encoded in the ATL follow a non-linear activation trajectory, as is often observed in deep network architectures, that is sufficient to make “early” and “late”

representations importantly different while still conveying aspects of the target structure (Rogers et al., 2021).

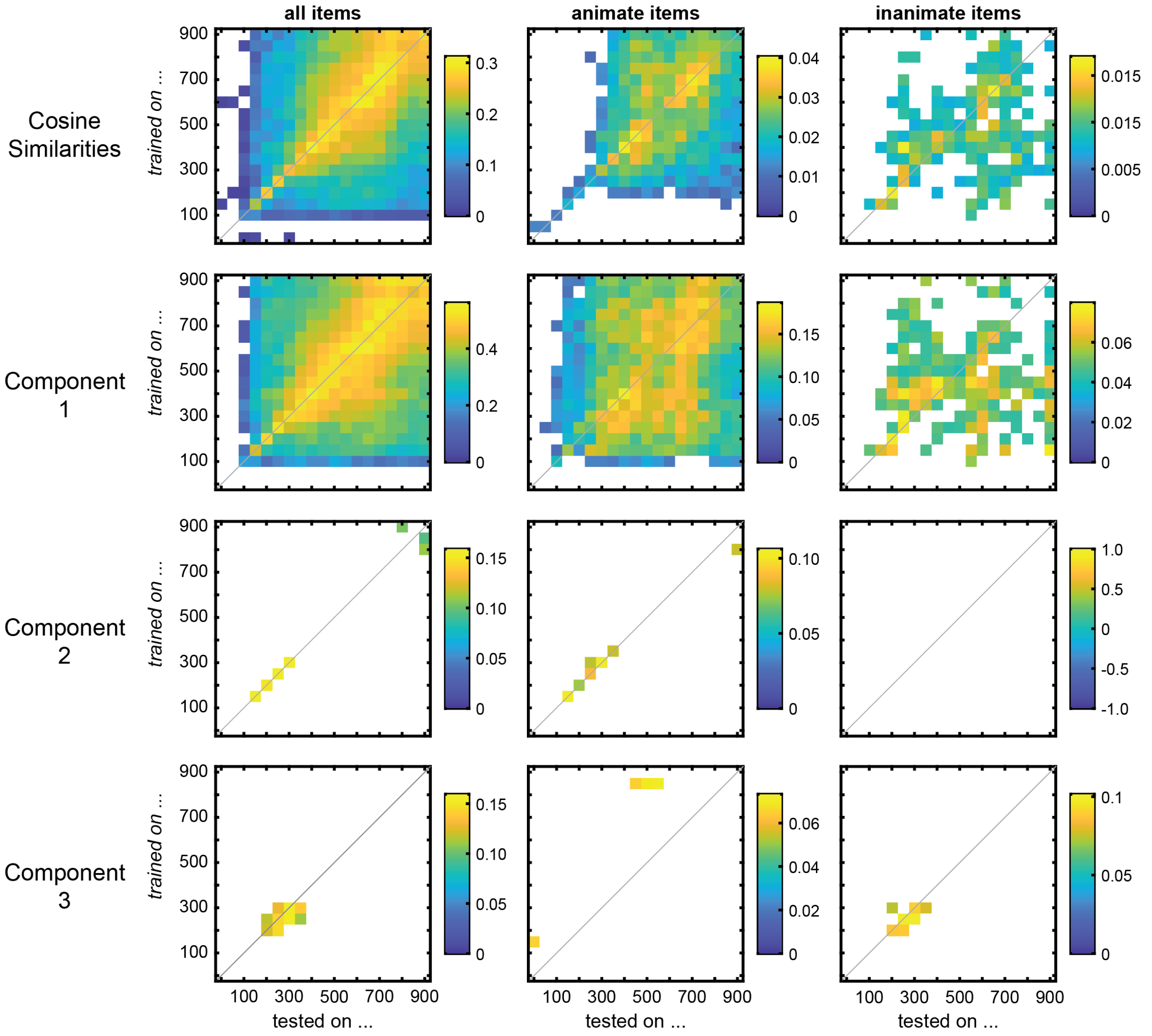

Figure S5. Temporal generalization profiles for each model fit within a single 100ms-moving-window and tested on all windows. Colored points are significant with FDR-corrected p<.05, correcting over the three panels in a single row (i.e., the multiple subsets relative to a shared dependent variable). The first row shows the correlations between predicted similarity matrices and the target similarity matrix. Subsequent rows show the correlation between single predicted and target components.

Supplement G: Anatomical distribution of decoding model weights across vATL

The decoding results reported in the main paper demonstrate that multidimensional semantic structure can be decoded from LFPs recorded from the vATL, and that decoding the second semantic dimension depends on regularization via the grOWL. This implies that local neural populations do not independently encode each semantic dimension, but simultaneously express multiple orthogonal components of the target semantic matrix. To evaluate whether this is so, we examined the spatial distribution of weights across electrodes in the decoding models predicting variation on the first via the second semantic dimensions for opening windows of size 200, 400, 600, 800, and 1000ms in the eight patients with electrodes implanted in the left hemisphere. To obtain a single number at each electrode for each model (each component is associated with a distinct model), the weight magnitudes (absolute values) were summed over time. Because the number of electrodes and their placement varied across participants, we projected these sums onto a standard MNI cortical surface and smoothed along the surface with a 4mm Gaussian kernel before averaging across participants. Points on the surface that are associated with data from fewer than six participants are excluded from analysis (i.e., cortical areas outside the red-line contour shown on surface plots in Figure 6a). To determine which sums are larger than expected by chance, the data for each participant within each window was fit to 1,000 random row-wise permutations of the target structure. Each of these solutions were projected onto the MNI surface as described above, resulting in 1,000 surface maps per participant. Then we sampled a map from each participant to simulate one “experiment”,

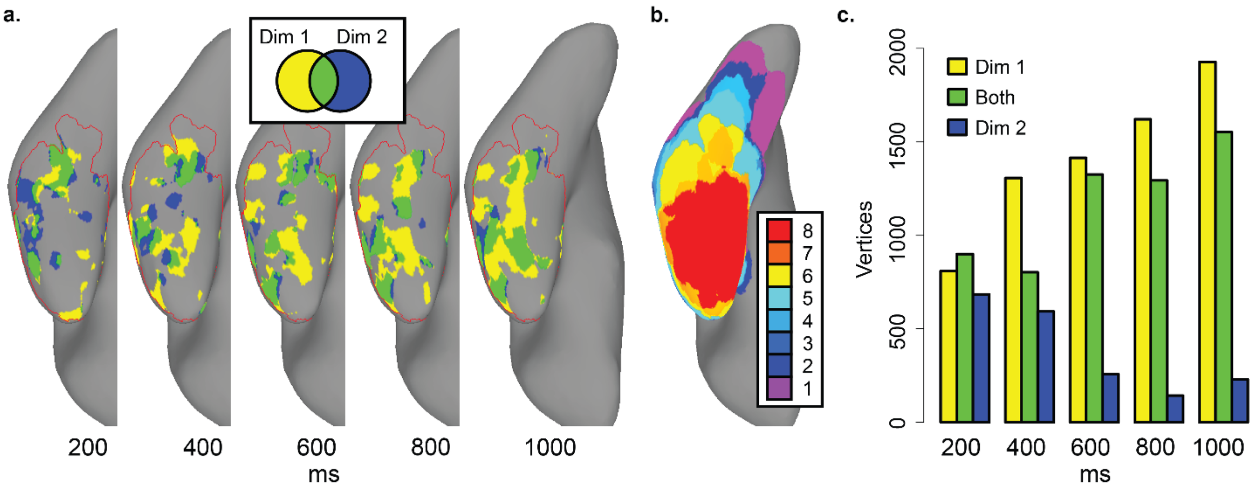

Figure S6. Significant vertices on the standard MNI N27 surface reconstruction displayed with AFNI SUMA for eight patients with left lateralized language (confirmed with the WADA test). a) RSL model coefficients (multi-task linear regression with grOWL regularization) associated with points in MNI space were projected onto and smoothed along the surface with a 4mm Gaussian kernel, separately for dimensions 1 and 2. This was repeated for 10,000 group-level maps simulating the null distribution at each vertex by permutation. Only points on the surface where data from 6 or more patients is available are considered; vertices meeting this criterion are contained within the red contour. Vertices with average weight magnitude larger than 9,500 of the 10,000 group-level values in the permutation distribution for that vertex are shown: points that meet these criteria for dimension 1 are shown in yellow, for dimension 2 are shown in blue, and for both are shown in green. b) Number of electrodes contributing to the data at each vertex after applying a 4mm Gaussian kernel. c) Tabulation of significant vertices.

and the data were summed and averaged as above. This was repeated 10,000 times to obtain a group-level null distribution (Stelzer et al., 2013).

Figure S6a displays the points on the cortical surface where the real average is larger than 9,500 of the values in this null distribution. Points on the surface that are associated with large model weights for the first component (but not the second) are shaded yellow; points that are associated with the second component (but not the first) are shown in blue. Points on the surface that are associated with large weights for both components in the same model are shown with green. Substantial overlap is observed in all windows, with overlap increasing with window length (**Figure S6c**). Both semantic dimensions are encoded across the vATL with little obvious functional localization, and the apparent contributions of a given area to each dimension appear to change depending on the size of the decoding window. The results are consistent with the view

that many vATL regions contribute to both dimensions of semantic structure, though they do not rule out the possibility that there exist small local regions independently encoding each dimension scattered across cortex.

Supplement H: Predicted embeddings

Representational similarity learning (RSL) involves decomposing a target representational similarity matrix (RSM) into orthogonal components (e.g., with singular value decomposition; SVD), composing a target embedding from a small selection of those components that explain most of the variance in the full matrix, and then training a linear model to decode each component in the target embedding. In our work the target embedding was three-dimensional (first three singular vectors of the RSM), and training and testing was performed with 10-fold cross validation such that every stimulus was a test item once. A model of the embedding determined with respect to the training set can be used to map spatiotemporal patterns of neural activity to predicted positions along each dimension of the embedding space (i.e., a coordinate referring to a point within the embedding space), including patterns associated with items assigned to the test set and therefore excluding from the training set. Test set predictions are obtained by weighting and then summing the neural features in the way that minimized the difference between predicted and target coordinates for *training set* items, and so reflect where the model expects these items to be located based on their neural pattern similarity with items in the training set.

By collecting these predicted coordinates for test set items across the 10 cross validation folds, we can visualize how each of the 100 items (50 animate, 50 inanimate) is positioned along each of the three target dimensions by models trained over different windows of time, averaged over the 10 participants. This is visualized in Figure S7. As expected, given the results reported in Figures 4 – 6 in the main document and the proportion of variance in the target RSM attributed to each dimension, separation of

animate from inanimate items along the first dimension is most salient in these predicted embeddings. This separation progresses over the first 300ms and remains relatively stable thereafter. Categories do not clearly separate along dimensions 2 or 3; there is variance along this dimension that systematically correlates with the target structure (Figure 4), but this presentation of predicted embeddings is not designed to convey how within-category variance (e.g., among land animals) is correlated with each target dimension. That there is stretching along dimensions 2 and 3, but not clear category separation, may reflect the smaller effect size of the within-domain correlations we report in the main paper, but is also consistent with the within-domain variance decoded from the ECoG signal being graded and continuous within sub-categories, not driven by ever-more minute category fractionization.

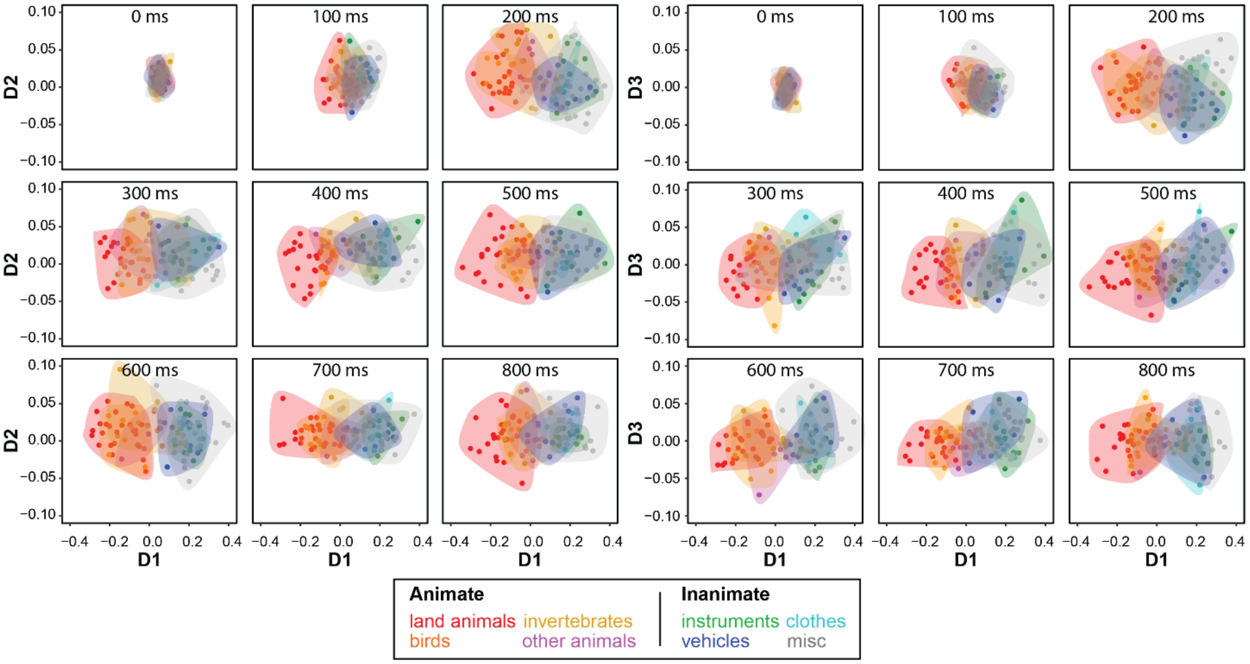

Figure S7. Predicted semantic embeddings for the 100 stimuli, averaged over 10 participants. Dots and shaded areas enclosing dots belonging to the same category are colored as in Figure 1. Which items belong to each category is reported in Supplement A, Table S1. Shaded areas were drawn by identifying the convex hull for the set of points and tracing the hull with a spline.

Supplemental References

Figueiredo, M., & Nowak, R. (2016). Ordered weighted l1 regularized regression with strongly correlated covariates: Theoretical aspects. Artificial Intelligence and Statistics,

Rogers, T. T., Cox, C. R., Lu, Q., Shimotake, A., Kikuchi, T., Kunieda, T., Miyamoto, S., Takahashi, R., Ikeda, A., Matsumoto, R., & Lambon Ralph, M. A. (2021). Evidence for a deep, distributed and dynamic code for animacy in human ventral anterior temporal cortex. *Elife*, *10*, e66276. https://doi.org/10.7554/eLife.66276

Stelzer, J., Chen, Y., & Turner, R. (2013). Statistical inference and multiple testing correction in classification-based multi-voxel pattern analysis (MVPA): random permutations and cluster size control. *NeuroImage*, *65*, 69-82. https://doi.org/10.1016/j.neuroimage.2012.09.063

Yuan, M., & Lin, Y. (2006). Model selection and estimation in regression with grouped variables. *Journal of the Royal Statistical Society: Series B (Statistical Methodology)*, *68*(1), 49-67. https://doi.org/10.1111/j.1467-9868.2005.00532.x
